# Basic CSF parameters and MRZ reaction help in differentiating MOG antibody-associated autoimmune disease versus multiple sclerosis

**DOI:** 10.3389/fimmu.2023.1237149

**Published:** 2023-09-06

**Authors:** Benjamin Vlad, Ina Reichen, Stephan Neidhart, Marc Hilty, Dimitra Lekaditi, Christine Heuer, Amanda Eisele, Mario Ziegler, Markus Reindl, Andreas Lutterotti, Axel Regeniter, Ilijas Jelcic

**Affiliations:** ^1^ Department of Neurology, University Hospital Zurich, Zurich, Switzerland; ^2^ Neuroimmunology and Multiple Sclerosis Research Section, Department of Neurology, University Hospital Zurich, Zurich, Switzerland; ^3^ Clinical Department of Neurology, Medical University of Innsbruck, Innsbruck, Austria; ^4^ Infectious Disease Serology and Immunology, Medica Medizinische Laboratorien Dr. F. Kaeppeli AG, Zurich, Switzerland

**Keywords:** MOGAD, multiple sclerosis, cerebrospinal fluid, MRZ reaction, oligoclonal bands, CSF/serum albumin ratio

## Abstract

**Background:**

Myelin oligodendrocyte glycoprotein antibody-associated autoimmune disease (MOGAD) is a rare monophasic or relapsing inflammatory demyelinating disease of the central nervous system (CNS) and can mimic multiple sclerosis (MS). The variable availability of live cell-based MOG-antibody assays and difficulties in interpreting low-positive antibody titers can complicate diagnosis. Literature on cerebrospinal fluid (CSF) profiles in MOGAD versus MS, one of the most common differential diagnoses, is scarce. We here analyzed the value of basic CSF parameters to i) distinguish different clinical MOGAD manifestations and ii) differentiate MOGAD from MS.

**Methods:**

This is retrospective, single-center analysis of clinical and laboratory data of 30 adult MOGAD patients and 189 adult patients with relapsing-remitting multiple sclerosis. Basic CSF parameters included CSF white cell count (WCC) and differentiation, CSF/serum albumin ratio (Q_Alb_), intrathecal production of immunoglobulins, CSF-restricted oligoclonal bands (OCB) and MRZ reaction, defined as intrathecal production of IgG reactive against at least 2 of the 3 viruses measles (M), rubella (R) and varicella zoster virus (Z).

**Results:**

MOGAD patients with myelitis were more likely to have a pleocytosis, a Q_Alb_ elevation and a higher WCC than those with optic neuritis, and, after review and combined analysis of our and published cases, they also showed a higher frequency of intrathecal IgM synthesis. Compared to MS, MOGAD patients had significantly more frequently neutrophils in CSF and WCC>30/µl, Q_Alb_>10×10^-3^, as well as higher mean Q_Alb_ values, but significantly less frequently CSF plasma cells and CSF-restricted OCB. A positive MRZ reaction was present in 35.4% of MS patients but absent in all MOGAD patients. Despite these associations, the only CSF parameters with relevant positive likelihood ratios (PLR) indicating MOGAD were Q_Alb_>10×10^-3^ (PLR 12.60) and absence of CSF-restricted OCB (PLR 14.32), whereas the only relevant negative likelihood ratio (NLR) was absence of positive MRZ reaction (NLR 0.00).

**Conclusion:**

Basic CSF parameters vary considerably in different clinical phenotypes of MOGAD, but Q_Alb_>10×10^-3^ and absence of CSF-restricted OCB are highly useful to differentiate MOGAD from MS. A positive MRZ reaction is confirmed as the strongest CSF rule-out parameter in MOGAD and could be useful to complement the recently proposed diagnostic criteria.

## 1 Introduction

Myelin oligodendrocyte glycoprotein antibody-associated autoimmune disease (MOGAD) is a rare inflammatory demyelinating disease of the central nervous system (CNS), which causes a broad spectrum of atypical, partly multiple sclerosis (MS)-mimicking demyelinating CNS syndromes including (recurrent) optic neuritis (ON), myelitis, aquaporin-4 (AQP4)-seronegative neuromyelitis optica (NMO)-like disease, (brainstem) encephalitis and others (1). It is considered a disease entity separate from MS and AQP4-seropositive NMO because of different immunological, histopathological, serological, clinical and paraclinical features, as well as distinct therapy responses and prognosis (2–5). The prevalence in Europe amounts to approximately 2/100.000 (6, 7), which makes its occurrence significantly rarer than MS (approximately 190/100.000) (8), but slightly more frequent than NMO (approximately 1/100.000) (9). While the ability to diagnose MOGAD has increased in recent years, distinguishing MOGAD from MS remains a challenge, as there are different live cell-based assays for detecting MOG-specific antibodies in serum (and to some extent in CSF) that appear to offer much higher specificity compared with commercial assays that use fixed cells expressing full-length MOG (10). Especially, low titers of MOG-specific antibodies are difficult to interpret correctly and may lead to false-positive or false-negative findings (11, 12). In contrast to MS, MOGAD can present with either monophasic or relapsing disease course (13, 14), but predicting which disease course is most likely to develop after the first relapse is not possible based on the current state of knowledge (15). Additionally, CSF parameters from routine clinical work up can differ considerably between the two entities and can already provide decisive clues for differentiating between both diseases (16), but concrete laboratory constellations and patterns of CSF findings for this purpose have been insufficiently described and validated to date. We aimed to analyze differences in demographic, clinical and CSF findings between MOGAD and MS on mono-center level, including the MRZ reaction (MRZR), which is defined as a polyspecific intrathecal production of IgG against ≥2 of 3 antigens, i.e. measles (M), rubella (R), and zoster (Z) virus and represents the most specific CSF biomarker for MS to date.

## 2 Materials and methods

### 2.1 Patients

We retrospectively analyzed demographic, clinical and laboratory data from 30 patients with MOGAD. None of the MOGAD patients received disease-modifying therapy before lumbar puncture. 28 MOGAD patients received lumbar puncture in relapse, but 2 patients received lumbar puncture in remission more than 30 days after steroid treatment at a peripheral center. Basic CSF parameters in MOGAD patients with monophasic and polyphasic disease course were compared. In a second step, basic demographic and CSF data from MOGAD patients and 189 patients with untreated relapsing-remitting MS were compared for CSF white cell count (WCC) and white cell differentiation, CSF and serum albumin with calculated ratio (Q_Alb_), CSF/serum ratios of IgG, IgA and IgM, respectively (Q_IgG_, Q_IgA_, Q_IgM_), frequency of intrathecal synthesis of IgG, IgA and IgM according to Reiber (17), respectively, frequency of CSF-restricted oligoclonal bands (OCB) and OCB patterns, intrathecal production of IgG reactive to measles (M), rubella, and varicella zoster (Z) viruses, called MRZ reaction (MRZR), CSF lactate levels and CSF/serum glucose ratio. Furthermore, basic CSF data from MOGAD and MS patients with (i) absence of CSF-restricted OCB and (ii) ON as first clinical event were compared. All MOGAD patients tested negative for AQP4-specific antibodies in serum and fulfilled the diagnostic criteria for MOGAD as recently proposed by the international MOGAD panel (15), i.e. patients were diagnosed with MOGAD based on MOGAD-typical clinical events such as unilateral- or bilateral optic neuritis or myelitis (**Table 1**), in most cases presence of radiological findings typical of MOGAD (such as bilateral simultaneous signal changes of the optic nerves, longitudinal optic nerve involvement [> 50% length of the optic nerve], perineural optic sheath enhancement, and/or optic disc oedema, or spinal cord signal changes compatible with longitudinally extensive myelitis extending over three or more vertebral segments, involving the conus, thoracolumbar radices and/or central cord or central grey matter as “H-sign” in axial sequences) and absence of radiological findings typical of MS as defined by Filippi et al. (18) and Wattjes et al. (19), and in all cases evidence of positive cell-based MOG antibody assay results in serum (see below). Other differential diagnoses such as MS, AQP4-seropositive neuromyelitis optica spectrum disease, neurosarcoidosis, neuro-Sjögren, CNS systemic lupus erythematosus and/or other autoimmune or infectious causes were excluded by means of clinical, radiological and/or laboratory findings.

**Table 1:** Demographic and clinical characteristics of MOGAD patients.

Disease course	
- Monophasic, n (%)	16/30 (53.3%)
- Relapsing, n (%)	14/30 (46.7%)
Follow-up time in months	
- All, median [Q1,Q3]	28.0 [10.0, 57.0]
- Monophasic disease course, median [Q1,Q3]	21.5 [7.5, 53.5]
- Relapsing disease course, median [Q1,Q3]	33.0 [15.0, 77.0]
First clinical event	
- Unilateral optic neuritis	18/30 (60.0%)
- Bilateral optic neuritis	3/30 (10.0%)
- Longitudinal extensive transverse myelitis	9/30 (30.0%)
Lumbar puncture	
- During first clinical event	28/30 (93.3%)
- after first clinical event, in remission	2/30 (6.7%)

All MS patients were diagnosed with relapsing-remitting MS and fulfilled the criteria for the diagnosis of MS according to the 2017 revised McDonald criteria (20), i.e. diagnosis of MS was based on the combination of typical clinical, radiological and CSF laboratory findings and exclusion of other differential diagnoses, including MOGAD. In most MS cases, MOGAD could be excluded by detection of MS-typical radiological findings and absence of MOGAD-typical findings. All patients with MS were untreated and had not received steroids before lumbar puncture, and lumbar puncture was in all cases performed during MS relapse. We retrospectively controlled, how many MOGAD patients fulfilled diagnostic criteria for MS, and how many MS patients fulfilled diagnostic criteria for MOGAD. Furthermore, all MOGAD and MS patients were checked for supporting MOGAD-typical radiological findings (15) and/or MS-typical radiological findings (18, 19).

Informed consent was obtained from all patients or relatives. Since data of all patients were anonymized for this study, the local Cantonal Ethics Committee stated that the research project does not fall within the scope of the Human Reseach Act (HRA) and therefore, an authorization from the ethics committee is not required (BASEC Nr. Req-2022-01134).

### 2.2 MOG-specific IgG testing

In case of clinical suspicion, patients were tested for MOG-specific IgG in the in-house laboratory with a commercial kit using a fixed cell-based assay (“Assay A”) (Euroimmun, Kriens, Switzerland) and/or at the Neurological Routine and Research Laboratory, Clinical Department of Neurology of the Medical University of Innsbruck (M. Reindl), which used a live cell-based assay (Live CBA-IF, IgG(H+L) + Fc) quantified by immunofluorescence and end-point titration (“Assay B”) (10). Cut-off titer for MOG antibody positivity was ≥1:10 in assay A and ≥1:160 in assay B, respectively. Cut-off titer for high-titer MOG antibody levels (“clear positive”) was ≥1:320 in assay A and ≥1:640 in assay B, respectively (10). In the case of weakly positive titers (≤1:320) or negative results in assay A and persistent suspicion of MOGAD, the samples were tested with assay B (**Supplementary Table 1**).

### 2.3 Cytological examination and clinical chemistry

At the CSF Laboratory of the Department of Neurology, University Hospital Zurich, cytological examinations of the CSF follow a standardized protocol as part of the clinical routine. This protocol follows the recommendations of the German Society of CSF Diagnostics and Clinical Neurochemistry (DGLN e.V.) and still represents the gold standard of cytological examination of the CSF, since automated analysis of CSF cells by flow cytometry or other automated devices are not optimized for the analysis of samples comprising low cell numbers, such as the CSF (21). Briefly, CSF-infiltrating cells of all CSF samples are counted using a Fuchs Rosenthal counting chamber under the microscope within 1 hour after lumbar puncture in order to determine WCC. If pleocytosis is detected, CSF-infiltrating cells are examined microscopically to differentiate physiologic CSF cells and search for abnormalities. For this purpose, CSF is centrifuged using cytospin preparations with cytofunnels and cytoclips (Thermo Scientific, Basel, Switzerland), and cytospin probes are stained with the standard May-Grünwald-Giemsa procedure. Approximately 200 cells are differentiated by experienced CSF cytologists microscopically and classified as lymphocytes, monocytes, plasma cells, neutrophils, eosinophils, basophils, and macrophages. Bone marrow cells, mitoses, cells lining the CSF space and other cell types are described separately. CSF cell counting and CSF cell differentiation is done by four experienced CSF cytologists as part of the clinical routine, where each sample is analyzed by one CSF cytologist and validated by another CSF cytologist. All four CSF cytologists are medical technical assistants, each with more than 10 years of experience in cytological examinations of CSF, and they are trained for CSF cell differentiation according to the recommendations of the German Society of CSF Diagnostics and Clinical Neurochemistry (DGLN e.V.) (22).

A WCC>4/µl was classified as increased, representing pleocytosis. WCC was further grouped into subgroups of 0-4/µl, 4-30/µl and >30/µl as a measurement of pleocytosis severity. In a subgroup of patients with pleocytosis, differentiation of CSF white cells into respective leukocyte subpopulations, and the frequency of plasma cells, neutrophils, eosinophils, basophils and macrophages were available from clinical routine and were used for retrospective analysis. An age-dependent cut-off was applied for CSF lactate level interpretation as described by Jarius et al. (23). CSF/blood ratio of glucose was calculated, and a ratio of <0.5 was considered pathologic.

### 2.4 Evaluation of blood-CSF barrier function and humoral immune response

Albumin, IgG, IgM and IgA levels in CSF and serum were quantified by immunonephelometry (Atellica NEPH 630 System, Siemens Healthineers, Switzerland) and their respective CSF/serum ratios calculated. Blood-CSF barrier function (BCSFB) was assessed using CSF/serum albumin quotient (Q_Alb_). The upper reference limit of Q_Alb_ (Q_lim_) was calculated as [4+(a/15)]×10^-3^ according to Reiber (24), with “a” representing the patient’s age. Dysfunction of the BCSFB was defined as Q_Alb_>Q_lim_.

IgG index was calculated as Q_IgG_/Q_Alb_, with Q_IgG_=CSF IgG concentration/serum IgG concentration. The relative intrathecal fraction of IgG, IgA and IgM, respectively (IgG_IF_, IgA_IF_ and IgM_IF_), was calculated according to Reiber (17). IgG_IF_, IgA_IF_ and/or IgM_IF_>0% indicated significant intrathecal synthesis of IgG, IgA and/or IgM, respectively. OCBs were detected by isoelectric focusing (IEF) on agarose gels and immunoblotting using IgG-specific antibodies and a semi-automated approach (Interlab G26, Alberta, Canada). OCB patterns were evaluated according to international consensus criteria (25): OCB pattern 1=no OCBs in CSF or Serum; OCB pattern 2=CSF-restricted OCBs; OCB pattern 3=identical bands in CSF and serum and additional CSF-restricted OCBs; OCB pattern 4=identical OCBs in CSF and serum; and OCB pattern 5=monoclonal bands in CSF and serum. Intrathecal IgG synthesis was indicated only by IEF patterns 2 and 3. OCBs were considered CSF-restricted, if ≥2 additional bands were detected in CSF compared to serum.

### 2.5 MRZ reaction

IgG antibodies against measles (M), rubella (R) and varicella zoster (Z) viruses were measured in paired CSF and serum samples, either with commercial ELISA kits and fully automated ELISA processing (Euroimmun Analyzer I, Euroimmun AG, Kriens, Switzerland) or ELISA kits from Virion/Serion (one point calibration) and fully automated ELISA processing (4-plate ELISA processing system DSX, Dynex Technologies, Inc./Ruwag Bettlach, Switzerland).

The virus-specific CSF/serum antibody index (CAI_spec_) was calculated according to Reiber (17). In short, CAI_spec_ was assessed as CAI_spec_=Q_spec_/Q_IgG_ (if Q_Lim_ (IgG)>Q_IgG_), or CAI_spec=_Q_spec_/Q_Lim_ (IgG), if Q_Lim_ (IgG)<Q_IgG_). The respective parameters were calculated as follows: Q_spec=_antigen-specific IgG_CSF_ [AU]/antigen-specific IgGserum [AU]; Q_IgG=_total IgG_CSF_ [mg/l]/total IgG_serum_ [mg/l]; Q_Lim_ (IgG)=0.93×(Q_Alb_^2 ^+ 6×10^−6^)^0.5^−1.7×10^−3^; Q_Alb_=Alb_CSF_ [mg/l]/Alb_serum_ [mg/l] (with Alb=albumin). Q_Lim_ (IgG) refers to the upper discrimination line of the hyperbolic reference range for the blood-derived IgG in CSF as zero intrathecal IgG synthesis. CAI_spec_≥1.5 indicated intrathecal synthesis of virus-specific antibodies. MRZR was interpreted as positive according to Reiber et al. (26), if polyclonal intrathecal production of antibodies against ≥2 of the 3 antigens measles (M), rubella (R), and zoster (Z), was detectable (27).

External quality assurance covering CSF-/serum- albumin, -IgG, -IgM, -IgA and -OCB as well as MRZ reaction have been performed every 3-6 months in round robin tests organized by INSTAND e.V. (Düsseldorf, Germany) and have always been passed during the period of assessment of CSF findings.

### 2.6 Statistics

Differences in age and disease duration were compared with the Kruskal-Wallis test. Differences in frequency of female gender, pleocytosis, respective white cell subpopulations, elevated Q_Alb_, intrathecal immunoglobulin synthesis according to Reiber (17), CSF-restricted OCB, elevated CSF lactate, pathologic CSF/serum glucose ratio and positive MRZR were compared with Fisher’s exact test. Differences in mean values of WCC, Q_Alb_, immunoglobulin CSF/serum ratios and intrathecal fraction of IgG were calculated using the Mann-Whitney U test after testing for normal distribution with the Shapiro-Wilk test. Sensitivity, specificity, positive likelihood ratio (PLR) and negative likelihood ratio (NLR) with 95% confidence intervals (95% CI) of CSF/serum parameters were analyzed to estimate their value in distinguishing MOGAD from MS.

## 3 Results

All MOGAD patients were tested for MOG antibodies, of which 3 patients were tested only with the fixed cell-based assay (“Assay A”), 17 patients were tested only with the live cell-based assay (“Assay B”) and 10 patients were tested with both assays (**Supplementary Table 1**; **Supplementary Figure 1**). Retrospective data of MOG antibody results was available from 97/189 MS patients, 89 results from assay A and 23 results from assay B. 30/30 (100.0%) MOGAD patients and 1/97 (1.1%) MS patients tested positive for MOG antibodies, but 18/30 (60.0%) MOGAD patients and none of the MS patients showed a clear positive (high-titer) MOG antibody result (**Supplementary Table 2**). The one MS patient with a positive MOG antibody finding had a positive low-titer MOG antibody result (1:32) in assay A and tested negative in the more sensitive assay B. Since this patient showed MS-typical MRI changes and fulfilled the diagnostic criteria for MS, but not for MOGAD, this patient was diagnosed with MS. Altogether, assay A showed a sensitivity of 82.4% and specificity of 98.9% for MOGAD versus MS in our cohort, and assay B showed a sensitivity of 100.0% and specificity of 100.0% (**Supplementary Table 2**). All MOGAD patients, but none of the MS patients fulfilled diagnostic criteria for MOGAD (15), and all MS patients, but none of the MOGAD patients fulfilled diagnostic criteria for MS (20) (**Supplementary Table 3**). 2 MOGAD patients with optic neuritis (ON) did not fulfill radiological criteria for MOGAD in first brain MRI, but fulfilled diagnostic criteria for MOGAD and showed clear positive MOG antibody titers. 3 MOGAD patients with longitudinally extensive transverse myelitis (LETM) fulfilled diagnostic criteria for MOGAD and showed MOGAD-typical radiological signs, but also fulfilled radiological, but not diagnostic, criteria for MS (inflammatory lesions with dissemination in time and space). 2 of those 3 patients showed no CSF-restricted OCB. None of the MS patients showed MOGAD-typical MRI findings (**Supplementary Table 3**).

First clinical events in MOGAD patients included unilateral ON (18/30, 60%), LETM (9/30, 30%) and bilateral ON (3/30, 10%) (**Table 1**). During the follow-up period, 53.3% showed a monophasic disease course with a median follow-up of 21.5 months (IQR 7.5-53.5), whereas 46.7% were polyphasic (median follow-up 33.0 months [IQR 15.0-77.0]) (**Table 1**). Basic CSF parameters did not vary between MOGAD patients with monophasic and polyphasic disease course (**Supplementary Table 4**). Basic CSF parameters in MOGAD patients with ON as first clinical presentation varied from those with LETM, but due to the small number of LETM patients, almost all results were not significant. The only exception was the mean WCC (9.8 [SD 20.0] vs. 55.7 [SD 60.3], p=0.004) and the frequency of WCC>100/µl (0/21 [0.0%] vs. 3/9 [33.3%], p<0.001), which was significantly higher in MOGAD patients with LETM than with ON (**Supplementary Table 5**). Previously, Jarius et al. (16) reported differences of basic CSF parameters between acute ON and acute myelitis of adult MOGAD patients and found significant differences for frequency of pleocytosis (18/53 [34.0%] vs. 46/54 [85.2%], p<0.001), of WCC>100/µl (1/52 [1.9%] vs. 17/54 [31.5%], p<0.001), of elevated CSF lactate (7/39 [17.9%] vs. 18/37 [48.6%], p=0.007) and of elevated Q_Alb_ (16/46 [34.8%] vs. 28/50 [56.0%], p=0.043), but not for frequency of intrathecal synthesis of IgG, IgA or IgM, respectively (**Supplementary Table 5**). When we combined all MOGAD patients from our cohort and the cohort described by Jarius et al. (16) and compared all parameters between ON and LETM (**Supplementary Table 5**), we could confirm a significantly higher frequency of pleocytosis, of WCC>100/µl, of elevated CSF lactate and of elevated Q_Alb_ in LETM, and in addition, discovered for the first time significant differences in frequency of intrathecal synthesis of IgM (combined ON 4/60 [6.7%] vs. combined LETM 12/47 [25.5%], p=0.012). Differences in intrathecal synthesis of IgG, as determined by Reiber diagram or detection of CSF-restricted OCB), or intrathecal synthesis of IgA remained non-significant in both single and combined analysis (**Supplementary Table 6**).

**Figure 1:**
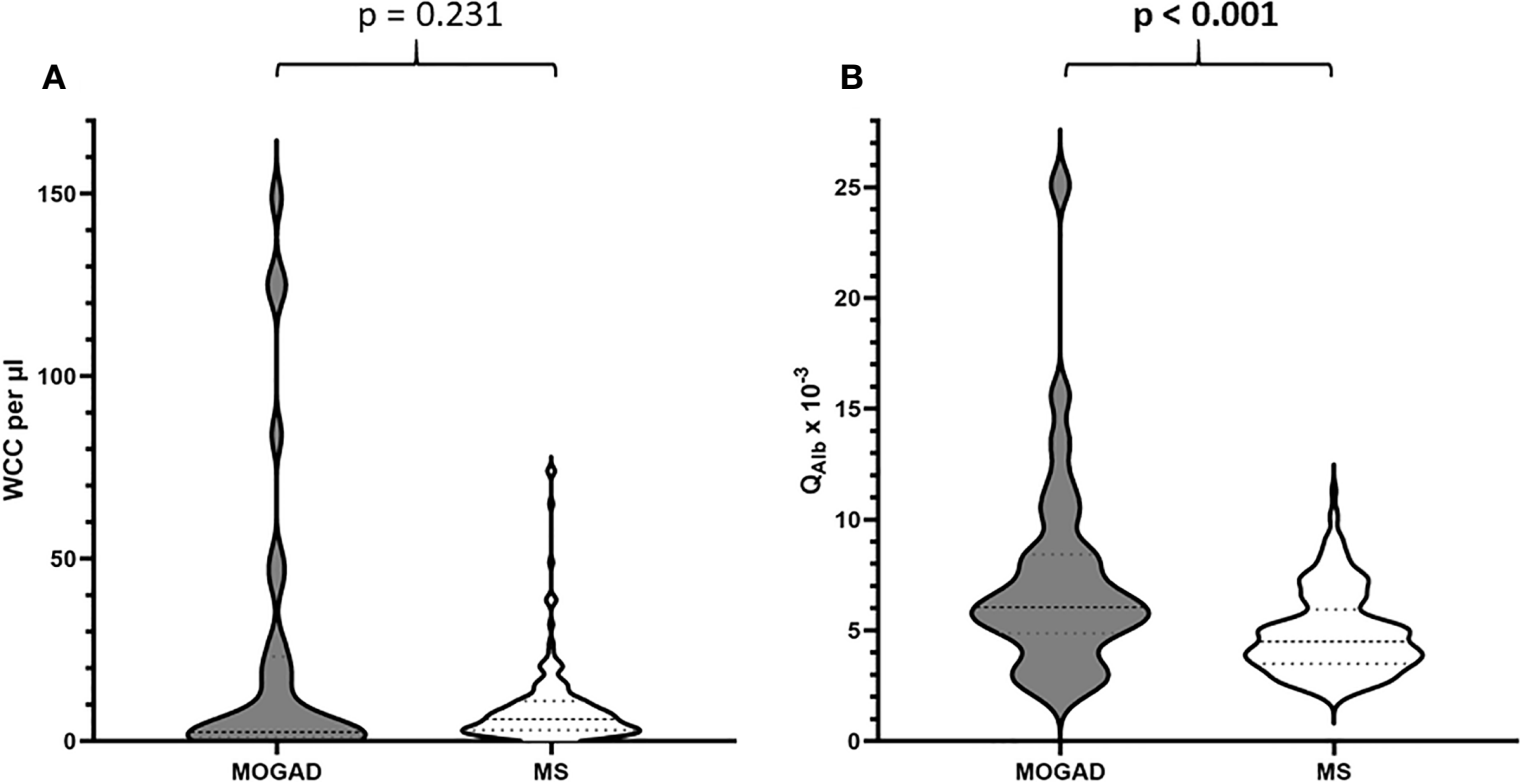
**(A)** Distribution of WCC per µl in patients with MOGAD vs. MS. **(B)** Distribution of Q_Alb_ x 10^-3^ in patients with MOGAD vs. MS.

**Figure 2:**
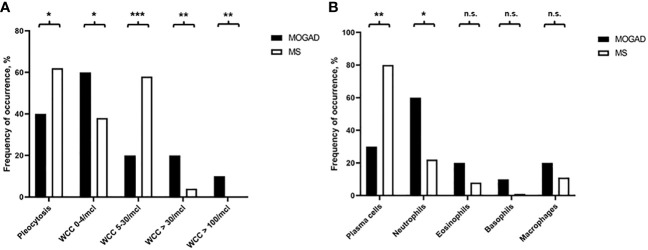
**(A)** Frequency of occurrence of pleocytosis, i.e. WCC 0-4/µl, WCC 5-30/µl, WCC>30/µl, or WCC>100/µl, in MOGAD and MS patients (%). **(B)** Frequency of occurrence of plasma cells, neutrophils, eosinophils, basophils, or macrophages in MOGAD and MS patients (%). n.s., not significant, * p<0.05, ** p<0.001, *** p<0.001.

In our cohort, patients with MOGAD were significantly older at time of lumbar puncture than MS patients (median age 40.5 [IQR 28.2-55.8] vs 31.0 [IQR 27.0-38.0] years, p=0.004), but did not differ in terms of disease duration (median 0.0 [IQR 0.0-2.5] vs 0.0 [IQR 0.0-2.0] months, p=0.773). Although MS patients tended to be more often female than MOGAD patients (64.6% vs. 46.7%), this finding was not statistically significant (p=0.094, **Table 2**). Pleocytosis was less frequent in MOGAD than in MS patients (40.0% vs. 61.9%, p=0.028), particularly in the WCC range 5-30/µl (20.0% vs. 57.7%, p<0.001), but mean WCC (in MOGAD 36.1/µl [SD 95.2/µl], in MS 9.1/µl [SD 10.9/µl], p=0.231) did not differ significantly (**Figure 1A**; **Supplementary Figure 2A**). Notably, WCC>30/µl was more frequent in MOGAD than in MS patients (20.0% vs. 4.2%, p=0.006) and WCC>100/µl occurred in MOGAD patients only (10.0% vs. 0.0%, p=0.002) (**Figure 2A**). In a subgroup of patients with pleocytosis (10 patients with MOGAD, 107 patients with MS), differentiation of CSF white cells was available (**Figure 2B**). While MS patients showed a significantly higher frequency of plasma cells (80.4% vs. 30.0%, p=0.002), MOGAD patients showed a significantly higher frequency of neutrophils (60.0% vs. 22.4%, p=0.018). Frequency of CSF eosinophils (20.0% vs. 8.4%, p=0.238), CSF basophils (10.0% vs. 0.9%, p=0.164) and CSF macrophages (20.0% vs. 11.2%, p=0.342) tended to be higher in MOGAD patients, but neither of those results were statistically significant (**Table 3**).

**Table 2:** Demographic features of MOGAD and MS patients.

Parameter	Overall	MOGAD	MS	p-value
N	219	30	189	-
Female gender, n (%)	136 (62.1%)	14 (46.7%)	122 (64.6%)	0.094
Age at LP, median [Q1,Q3]	32.0 [27.0, 40.0]	40.5 [28.2, 55.8]	31.0 [27.0, 38.0]	**0.004**
Disease duration in months, median [Q1,Q3]	0.0 [0.0, 2.0]	0.0 [0.0, 2.5]	0.0 [0.0, 2.0]	0.773

Bold values indicate statistical significance (p < 0.05).

**Table 3:** Comparison of A) CSF white cell counts and B) white cell differentiation in MOGAD and MS.

CSF parameter	Overall	MOGAD	MS	p-value
A) CSF WCC				
Pleocytosis, n/N (%)	129/219 (58.9%)	12/30 (40.0%)	117/189 (61.9%)	**0.028**
- WCC 0-4/μl, n/N (%)	90/219 (41.1%)	18/30 (60.0%)	72/189 (38.1%)	**0.028**
- WCC 5-30/μl, n/N (%)	115/219 (52.5%)	6/30 (20.0%)	109/189 (57.7%)	**<0.001**
- WCC>30/μl, n/N (%)	14/219 (6.4%)	6/30 (20.0%)	8/189 (4.2%)	**0.006**
- WCC>100/μl, n/N (%)	3/219 (1.4%)	3/30 (10.0%)	0/189 (0.0%)	**0.002**
CSF WCC, mean (SD)	12.8 (37.3)	36.1 (95.2)	9.1 (10.9)	0.231
B) CSF white cell differentiation*				
- Frequency of plasma cells, n/N (%)	89/117 (76.1%)	3/10 (30.0%)	86/107 (80.4%)	**0.002**
- Frequency of neutrophils, n/N (%)	30/117 (25.6%)	6/10 (60.0%)	24/107 (22.4%)	**0.018**
- Frequency of eosinophils, n/N (%)	11/117 (9.4%)	2/10 (20.0%)	9/107 (8.4%)	0.238
- Frequency of basophils, n/N (%)	2/117 (1.7%)	1/10 (10.0%)	1/107 (0.9%)	0.164
- Frequency of macrophages, n/N (%)	14/117 (12.0%)	2/10 (20.0%)	12/107 (11.2%)	0.342

*assessed in 117 patients (10 patients with MOGAD, 107 patients with MS) with WCC>4/μl. Bold values indicate statistical significance (p < 0.05).

**Figure 3:**
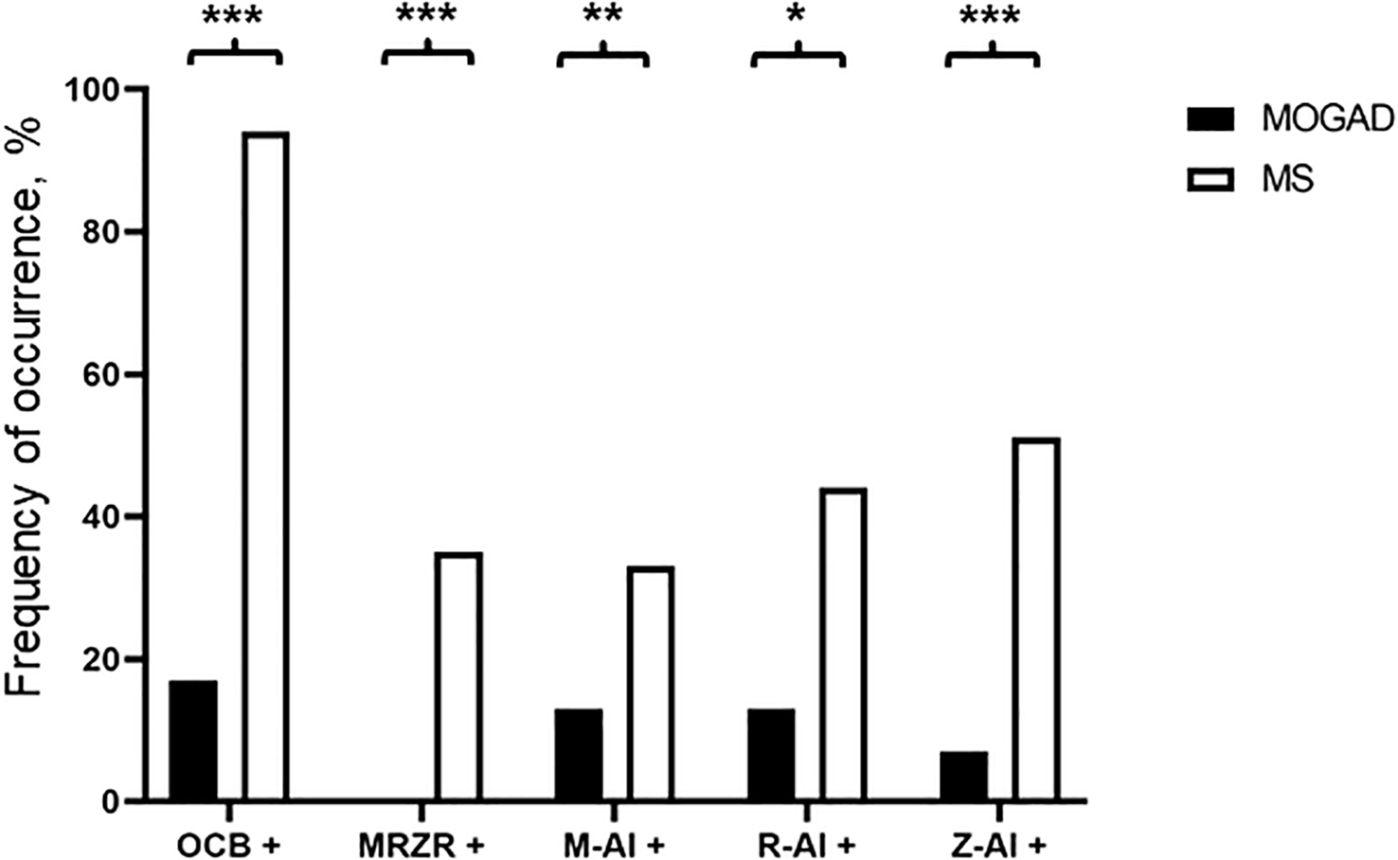
Frequency of occurrence of CSF-restricted OCB (OCB+), positive MRZR (MRZ+), positive M-CAI (M+), positive R-CAI (R+), and positive Z-CAI (Z+) in MOGAD and MS patients (%). * p<0.05, ** p<0.001, *** p<0.001.

Elevation of Q_Alb_ was more common in MOGAD than in MS (43.3% vs. 24.3%, p=0.044). An intermediate elevation of Q_Alb_>10×10^-3^ showed an even stronger association with MOGAD (16.7% vs. 1.6%, p=0.002) and mean Q_Alb_ was significantly higher in MOGAD than in MS patients (7.3 [SD 4.6] vs. 4.9 [SD 1.8], p<0.001) (**Figure 1B**; **Supplementary Figure 2B**). Mean CSF/serum IgG ratio (4.6 [SD 4.4] vs. 4.4 [SD 2.5], p=0.817) and mean CSF/serum IgM ratio (1.3 [SD 2.5] vs. 0.8 [SD 1.4], p=0.337) showed no significant differences between both groups, but there was a significant difference in mean CSF/serum IgA ratio (2.5 [SD 2.7] vs. 1.7 [SD 1.7], p=0.024) (**Supplementary Table 6**). Intrathecal synthesis of IgG according to Reiber (17) was less frequent in MOGAD than in MS patients (13.3% vs. 52.9%, p<0.001), whereas there was no significant difference in intrathecal synthesis of IgA (0.0% vs. 7.9%, p=0.233) and IgM (13.3% vs. 17.5%, p=0.794) (**Table 4**). If intrathecal IgG synthesis according to Reiber (17) was present, mean intrathecal fraction of IgG, i.e. IgG_IF_, did not vary between MOGAD and MS patients (31.1 [SD 23.9] vs. 38.8 [SD 18.4], p=0.617) (**Supplementary Table 6**). CSF-restricted OCB were present in most of the MS patients (94.2%), but only in 16.7% of the MOGAD patients (p<0.001) (**Figure 3**). Patients with MOGAD were furthermore significantly more likely to show pathological levels of CSF lactate (10.7% vs. 1.6%, p=0.030) and pathological CSF/serum glucose ratio (14.3% vs. 0.0%, p<0.001) than MS patients (**Table 4**). Of particular interest, a positive MRZR was only found in MS patients and in none of MOGAD patients (35.4% vs. 0.0%, p<0.001, **Table 5**; **Figure 3**). 6/30 (20.0%) MOGAD patients had a single virus-specific antibody reactivity (**Supplementary Table 7**). Rubella- and zoster-specific CAI values, but not measles-specific CAI values, were significantly lower in MOGAD patients than in MS patients (**Supplementary Table 8**).

**Table 4:** Comparison of basic CSF parameters in MOGAD and MS.

CSF parameter	Overall	MOGAD	MS	p-value
Elevated Q_Alb_, n/N (%)	59/219 (26.9%)	13/30 (43.3%)	46/189 (24.3%)	**0.044**
- Q_Alb_>10×10^-3^	8/219 (3.7%)	5/30 (16.7%)	3/189 (1.6%)	**0.002**
- Q_Alb_, mean (SD)	5.2 (2.5)	7.3 (4.6)	4.9 (1.8)	**<0.001**
IgG_IF_>0%, n/N (%)	104/219 (47.9%)	4/30 (13.3%)	100/189 (52.9%)	**<0.001**
IgA_IF_>0%, n/N (%)	15/219 (6.8%)	0/30 (0.0%)	15/189 (7.9%)	0.233
IgM_IF_>0%, n/N (%)	37/219 (16.9%)	4/30 (13.3%)	33/189 (17.5%)	0.794
CSF-restricted OCB, n/N (%)	183/219 (83.6%)	5/30 (16.7%)	178/189 (94.2%)	**<0.001**
Elevated CSF lactate, n/N (%)	6/215 (2.8%)	3/28 (10.7%)	3/187 (1.6%)	**0.030**
Pathologic CSF/serum glucose ratio, n/N (%)	4/215 (1.9%)	4/28 (14.3%)	0/187 (0.0%)	**<0.001**

Bold values indicate statistical significance (p < 0.05).

**Table 5:** Frequency of intrathecal IgG production against measles (M)-, rubella (R)- or zoster (Z) antigens and of positive MRZ reaction in MOGAD and MS.

CSF parameter	Overall	MOGAD	MS	p-value
Intrathecal measles-specific IgG production (M), n/N (%)	67/219 (30.6%)	4/30 (13.3%)	63/189 (33.3%)	**0.032**
Intrathecal rubella-specific IgG production (R), n/N (%)	88/219 (40.2%)	4/30 (13.3%)	84/189 (44.4%)	**0.001**
Intrathecal zoster-specific IgG production (Z), n/N (%)	98/219 (44.7%)	2/30 (6.7%)	96/189 (50.8%)	**<0.001**
Positive MRZ reaction^1^, n/N (%)	67/219 (30.6%)	0/30 (0.0%)	67/189 (35.4%)	**<0.001**

^1^positive MRZ reaction (MRZR) was defined as intrathecal production of IgGs reactive against at least two of three antigens, measles (M), rubella (R) and varicella zoster (Z) virus antigens, i.e, M+R or M+Z or R+Z or M+R+Z. Bold values indicate statistical significance (p < 0.05).

Our findings of increased frequency of pleocytosis and BCSFB dysfunction in MOGAD patients, as well as decreased frequency of CSF-specific OCBs are in line with the results reported in the current literature (13, 16, 23, 28–30) (**Supplementary Table 9**). Interestingly, until now, a positive MRZR has been reported in 0/62 samples of 48 adult MOGAD patients (16), 0/28 samples of 24 pediatric MOGAD patients (23) and 0/30 patients in our study (**Supplementary Table 10**), resulting in the absence of a positive MRZR as a typical finding in MOGAD.

The probable prevalence of MOG seropositivity and positive MRZR in the general population can be calculated according to the estimated population-based prevalence of the respective disease (2/100.000 for MOGAD, 190/100.000 for MS) (6–8) and the disease-specific sensitivity of the respective positive diagnostic test. With a MOG seropositivity in 100% of MOGAD patients and 35.4% of MS patients being positive MRZR in our cohort, the estimated population-based prevalence of MOG seropositivity is more than 33x less frequent than a positive MRZR (2/100.000 vs. 67.3/100.000, p<0.00001) (**Supplementary Table 11**). The odds ratio for a positive disease-specific test result is 33.5 (95% CI 8.2-136.8, p<0.00001), when positive MRZ reaction is compared to MOG seropositivity and all true MS and MOGAD patients are included for testing. Based on these numbers, testing MOG antibodies in patients with low probability of MOGAD, e.g. including all MS-caused ON and myelitis cases, would result in a significant number of false-positive MOG antibody results in patients with low positive MOG serotiters (**Supplementary Table 12**).

In order to determine the value of single CSF parameters, sensitivity and specificity as well as positive and negative likelihood ratios (PLR and NLR) were calculated (**Table 6**). A PLR>10 and NLR<0.1 are considered useful in general practice (31, 32). Highest sensitivity for MOGAD was found in absence of intrathecal IgG synthesis (86.6% according to Reiber diagram, 83.3% according to IEF) and absence of a positive MRZR (100.0%), whereas reduced CSF/serum glucose ratio (100.0%), elevated CSF lactate (98.4%), Q_Alb_>10×10^-3^ (98.4%), WCC>30/µl and absence of CSF-restricted OCB (94.2%) showed highest specificity for MOGAD. Highest PLRs for MOGAD were found for absence of CSF-restricted OCB (14.32) and Q_Alb_>10×10^-3^ (12.60), while the potentially highest PLR for MOGAD, i.e. reduced CSF/serum glucose ratio, could not be calculated due to a specificity of 100.0%. The by far lowest NLR for MOGAD was found for absence of positive MRZR (0.00), followed by absence of CSF-restricted OCB (0.18) and absence of intrathecal IgG synthesis according to Reiber diagram (0.25). In addition, combined analysis of multiple parameters, i.e. i) WCC>30/µl or absence of CSF-restricted OCB, and simultaneous absence of positive MRZR, or ii) Q_Alb_>10×10^-3^ or absence of CSF-restricted OCB, and simultaneous absence of positive MRZ reaction, showed a significant PLR (12.60) with an overall low NLR (0.14), the second lowest NLR value detected (**Table 7**).

**Table 6:** Sensitivity, specificity, and likelihood ratios of single basic CSF parameters to distinguish MOGAD from MS.

CSF parameters	MOGAD	MS	Sensitivity (95% CI)	Specificity (95% CI)	PLR (95% CI)	NLR (95% CI)
1. Pleocytosis	12/30 (40.0%)	117/189 (62.0%)	40.0% (24.6-57.7)	38.1% (31.5-45.2)	0.65 (0.4-1.02)	1.58 (1.12-2.22)
2. WCC>30/μl	6/30 (20.0%)	8/189 (4.0%)	20.0% (9.3-37.8)	95.8% (91.7-97.9)	4.73 (1.76-12.66)	0.84 (0.70-1.00)
3. Presence of plasma cells	3/10 (30.0%)	86/107 (80.0%)	30.0% (10.6-60.8)	19.6% (13.2-28.3)	0.37 (0.14-0.97)	3.57 (2.04-6.23)
4. Presence of neutrophils	6/10 (60.0%)	24/107 (22.0%)	60.0% (31.2-83.1)	77.6% (86.76-84.5)	2.68 (1.44-4.96)	0.52 (0.24-1.11)
5. Elevated Q_Alb_	13/30 (43.0%)	46/189 (24.3%)	43.0% (27.4-60.8)	75.7% (69.0-81.2)	1.78 (1.10-2.88)	0.75 (0.54-1.04)
6. Q_Alb_>10×10^-3^	6/30 (20.0%)	3/189 (1.6%)	20.0% (9.3-37.8)	98.4% (95.2-99.7)	**12.60** (3.33-47.70)	0.81 (0.68-0.97)
7. Absence of IgG_IF_>0%	26/30 (86.6%)	89/189 (47.1%)	86.6% (69.5-95.2)	52.9% (45.8-59.9)	1.84 (1.50-2.26)	0.25 (0.10-0.63)
8. Absence of CSF-spec. OCB	25/30 (83.3%)	11/189 (5.8%)	83.3% (65.8-93.0)	94.2% (89.7-96.8)	**14.32** (7.89-25.97)	0.18 (0.08-0.39)
9. Absence of positive MRZR	30/30 (100.0%)	122/189 (64.6%)	100.0% (86.2-100.0)	35.4% (29.0-42.5)	1.55 (1.39-1.72)	**0.00** **(-)**
10. CSF lactate elevated	3/28 (10.7%)	3/187 (1.6%)	10.7% (3.0-28.2)	98.4% (95.1-99.6)	6.68 (1.42-31.47)	0.91 (0.80-1.03)
12. CSF/serum glucose ratio reduced	4/28 (14.3%)	0/187 (0.0%)	14.3% (5.2-32.3)	100.0% (97.5-100.0)	–	0.86 (0.74-1.00)

Bold values indicate statistical significance (p < 0.05).

**Table 7:** Sensitivity, specificity, and likelihood ratios of combinations of basic CSF parameters to distinguish MOGAD from MS.

Combination of CSF parameters	MOGAD	MS	Sensitivity (95% CI)	Specificity (95% CI)	PLR (95% CI)	NLR (95% CI)
1. Combination of **a)** 1 of the following parameters: - WCC>30/μl - or absence of CSF-restricted OCB and **b)** absence of positive MRZ reaction	26/30 (86.7%)	13/189 (6.9%)	86.7% (69.5-95.2)	93.1% (88.5-96.0)	**12.60** (7.32-21.69)	0.14 (0.06-0.36)
2. Combination of **a)** 1 of the following parameters: - Q_Alb_>10×10^-3^ - or absence of CSF-restricted OCB and **b)** absence of positive MRZ reaction	26/30 (86.7%)	13/189 (6.9%)	86.7% (69.5-95.2)	93.1% (88.5-96.0)	**12.60** (7.32-21.69)	0.14 (0.06-0.36)

Bold values indicate statistical significance (p < 0.05).

Despite some clear trends, the comparison of basic CSF parameters between MOGAD patients and MS patients without CSF-restricted OCB (n=25 vs. n=11) showed no significant differences in terms of mean WCC (24.7 [SD 45.7] vs. 3.1 [SD 2.7], p=0.564), frequency of pleocytosis (36.0% vs. 18.2%, p=0.439), WCC>15/µl (28.0% vs. 0.0%, p=0.076), WCC>30/µl (20.0% vs. 0.0%, p=0.295), elevated Q_Alb_ (44.0% vs. 18.2%, p=0.259), intrathecal synthesis of IgG (0.0% vs. 0.0%), IgA (0.0% vs. 0.0%) or IgM (8.0% vs. 9.1%, p=1.000), elevated CSF lactate (17.4% vs. 9.1%, p=1.000) or pathologic CSF/serum glucose ratio (13.0% vs. 0.0%, p=0.536) (**Supplementary Table 13**).

When comparing MOGAD and MS patients with ON as first clinical event (n=21 vs. n=74), mild pleocytosis was less frequent in MOGAD patients (28.6% vs. 55.4%, p=0.047), but higher mean WCC (9.8 [SD 20.0] vs. 8.4 [SD 11.2], p=0.017) and moderately increased pleocytosis (WCC>30/µl) was associated with MOGAD (19.0% vs. 4.1%, p=0.041). In addition, pathologic CSF/serum glucose ratio occurred in MOGAD patients only (10.5% vs. 0.0%, p=0.041). Intrathecal synthesis of IgG, either according to Reiber diagram (14.3% vs. 55.4%, p=0.001) or through presence of CSF-restricted OCB (14.3% vs. 93.2%, p<0.001), and positive MRZR (0.0% vs. 35.1%, p<0.001) proved useful to distinguish MOGAD from MS (**Table 8**). There was no statistical significance in terms of Q_Alb_ elevation (28.6% vs. 24.3%, p=0.777), intrathecal synthesis of IgA (0.0% vs. 8.1%, p=0.333) or IgM (4.8% vs. 12.2%, p=0.450) and elevated CSF lactate (5.3% vs. 0.0%, p=0.369).

**Table 8:** Comparison of basic CSF parameters in MOGAD and MS patients with optic neuritis as first clinical event.

CSF parameter	Overall	MOGAD	MS	P-value
Pleocytosis, n/N (%)	47/95 (49.8%)	6/21 (28.6%)	41/74 (55.4%)	**0.047**
WCC, mean (SD)	8.7 (13.5)	9.8 (20.0)	8.4 (11.2)	**0.017**
WCC>15/μl, n/N (%)	14/95 (14.7%)	4/21 (19.0%)	10/74 (13.5%)	0.503
WCC>30/μl, n/N (%)	7/95 (7.4%)	4/21 (19.0%)	3/74 (4.1%)	**0.041**
Elevated Q_Alb_, n/N (%)	24/95 (25.3%)	6/21 (28.6%)	18/74 (24.3%)	0.777
IgG_IF_>0%, n/N (%)	44/95 (46.3%)	3/21 (14.3%)	41/74 (55.4%)	**0.001**
IgA_IF_>0%, n/N (%)	6/95 (6.3%)	0/21 (0.0%)	6/74 (8.1%)	0.333
IgM_IF_>0%, n/N (%)	10/95 (10.5%)	1/21 (4.8%)	9/74 (12.2%)	0.450
CSF-restricted OCB, n/N (%)	72/95 (75.8%)	3/21 (14.3%)	69/74 (93.2%)	**<0.001**
Positive MRZR, n/N (%)	26/95 (27.4%)	0/21 (0.0%)	26/74 (35.1%)	**<0.001**
Elevated CSF lactate, n/N (%)	2/92 (2.2%)	1/19 (5.3%)	1/74 (1.4%)	0.369
Pathologic CSF/serum glucose ratio, n/N (%)	2/93 (2.2%)	2/19 (10.5%)	0/73 (0.0%)	**0.041**

Bold values indicate statistical significance (p < 0.05).

## 4 Discussion

In clinical routine, physicians find themselves daily in the situation of having to distinguish differential diagnoses in order to quickly initiate an indicated therapy. Autoimmune and inflammatory diseases of the central nervous system can clinically and radiologically present in a very similar fashion (33–36) at first manifestation, which is why a well thought-out strategy and interpretation of diagnostic findings is necessary. One of the most common differential diagnoses of multiple sclerosis, which is the most frequent chronic-inflammatory CNS disease (37), is MOGAD, for which diagnostic criteria have recently been proposed in a comprehensive consensus paper (15). In this retrospective single-center study, we demonstrate the importance of CSF routine diagnostics in clinical practice and describe the usefulness of absence of the biomarker MRZR, which is considered the most specific marker for multiple sclerosis to date (38), in distinguishing MOGAD from MS. Our results confirm the absence of a positive MRZR as a typical finding in MOGAD and add evidence to the data supporting positive MRZR as an MS-specific marker (38). Absence of positive MRZR could potentially complement the recently proposed diagnostic criteria of MOGAD (15). Since the diagnosis of MOGAD can be difficult due to, among other things, the clinical variability, the limited availability of high-sensitivity live cell-based assays (10), and the difficulty in interpreting low-titer antibody results, it is important to reliably distinguish between these two diseases and to identify patients who should be tested for MOG antibodies in the first place in order to avoid false-positive results by over-testing (39). Despite high sensitivities and specificities of MOG antibody assays of 95-100% (10), testing MOG antibodies in patients with low probability of MOGAD, e.g. including all MS-caused ON and myelitis cases, leads to a significant occurrence of false-positive test results and is therefore not recommended (10, 15, 39). As presented above, the estimated frequency of positive MRZR in the general population is more than 33 times higher than the estimated frequency of MOG seropositivity, while there are no reported cases of positive MRZR in MOGAD until now. Accordingly, if the pretest probability for MOG seropositivity is low, testing for MRZR should be favored over testing for MOG antibodies, and testing for MOG antibodies should be avoided if MRZR is positive.

MOGAD can occur both as a monophasic and relapsing-remitting disease (13, 14), but so far, there are no tools to predict the course at the time of diagnosis. Parameters to determine the course of the disease at an early stage would be desirable so that either an adequate immunomodulatory therapy can be initiated at an early stage, or a continuous immunomodulatory therapy is not started unnecessarily or given for too long time, respectively. In our cohort, routine CSF diagnostics did not appear useful in this regard, as there were no significant differences in terms of WCC, Q_Alb_ or intrathecal synthesis of immunoglobulins The analysis is limited by the low number of patients, variable follow-up time and different treatment strategies after the first relapse. Therefore, further work is needed to verify this in a bigger cohort.

The number of studies analyzing typical CSF profiles in MOGAD patients is low (13, 16, 23, 28–30) and most studies focus on pediatric MOGAD. The most relevant multicenter study for adult MOGAD patients involved 163 lumbar punctures in 100 adult MOGAD patients (16). Especially the absence of CSF-restricted OCB in the majority of samples (present in 19/150, 12.7%) and the absence of a positive MRZR in all patients (present in 0/48, 0.0%) were considered remarkable. The cellular immune response and function of the BSCFB varied widely within the cohort and was dependent on relapse and remission as well as the initial clinical manifestation. Of particular interest, in a notable proportion of samples, there was a moderate cell count increase above 50/µl (in 30/157, 19.1%) and 100/µl (in 19/157, 12.1%), which is considered a red flag for the diagnosis of MS, since only 5% of MS patients are found with CSF WCC>30/µl (25, 40). Regarding cell differentiation, the presence of neutrophils in 33/77 (42.9%) was particularly striking, whereas the presence of plasma cells (3/77, 3.9%) was rare. In about half of the samples, a dysfunction of the BSCFB could be detected (67/139, 48.2%) and a moderate dysfunction (defined as Q_Alb_>10×10^-3^) as well as a severe dysfunction (defined as Q_Alb_>20×10^-3^) occurred in a notable number of patients (exact number not stated). These results also appear useful to distinguish MOGAD from MS, as the latter has intact barrier function in approximately 90% of cases and elevation of Q_Alb_>10×10^-3^ is an exception (25, 40). A systematic analysis to assess the usefulness of these CSF parameters, either as single parameter or in combination, to distinguish MOGAD from MS in clinical practice has not yet been conducted.

It should be noted that the vast majority of our MOGAD patients had optic neuritis as first clinical presentation, which probably influenced the comparison, as MOGAD patients with acute optic neuritis have been shown to differ significantly from patients with acute myelitis in terms of WCC, frequency of pleocytosis, BSCFB dysfunction and even CSF-restricted OCB (16). Due to the small number of patients with myelitis, our analysis of differences between MOGAD patients with myelitis and ON was significantly limited, but a combined analysis of our work with the previously published cohort of Jarius et al. (16) not only confirms significant differences in frequency of pleocytosis, WCC>100/µl, BCSFB dysfunction and CSF lactate elevation, but revealed for the first time significant differences in the frequency of intrathecal synthesis of IgM. This could have implications for the understanding of MOGAD pathophysiology, as e.g. intrathecal IgM synthesis in MS is associated with spinal cord manifestation and with early activation of the complement cascade (41). BCSFB dysfunction has also been observed more often in MS patients with spinal lesions as compared to MS patients with supra- and/or infratentorial lesions (42), and Reiber (43) postulated that this could reflect reduced CSF- or interstitial fluid flow due to spinal lesions.

Jarius and colleagues (16) also showed CSF findings of the first-ever lumbar puncture in a subcohort, which corresponds to the diagnostic situation in our work and is suitable for comparing the data with our results. The majority of our MOGAD patients, especially if presenting with ON, had normal WCC (12/30 [40.0%)] vs. 55.7% [30.8% if ON] in Jarius et al. (16)), but the level of WCC varied widely between patients with pleocytosis. Q_Alb_ also varied widely, an elevation of Q_Alb_ was detected in 13/30 of our MOGAD cases (43% vs. 53.8% [42.4% if ON] in Jarius et al. (16)). In 5/30 of our MOGAD patients CSF-restricted OCB could be detected (16.7% vs. 9.6% in Jarius et al. (16)) and positive MRZR did not occur in either cohort. Regarding CSF white cell differentiation, our data is limited by the fact that only data from patients with pleocytosis were available and therefore the number of MOGAD patients with available data was very low (n=10), but our work confirms the increased occurrence of neutrophils and the less frequent occurrence of plasma cells in the CSF of MOGAD patients.

In order to determine their value, the PLR and NLR of single and combinational multiple parameters were calculated, and a PLR>10 and NLR<0.1 was considered meaningful (31, 32). There is a general lack of data on these ratios for diagnostic tests (32), but they are considered superior to sensitivity and specificity for clinical routine (44). Only a Q_Alb_>10×10^-3^ (PLR=12.60) and the absence of CSF-restricted OCB (PLR=14.32) showed a useful PLR (defined as PLR>10) and the absence of a positive MRZR (NLR=0.00) showed a useful NLR (defined as NLR<0.1) to distinguish MOGAD from MS. Notably, the PLR for a decreased CSF/serum glucose ratio could not be calculated because of its specificity of 100%. These results confirm the importance of MRZR as the most specific biomarker for MS. However, in clinical routine and especially in cases with high diagnostic uncertainty, it is not the individual parameters, but patterns of several findings that are considered for diagnosis. Looking at combinations of several parameters in patients with absence of a positive MRZR, both the combination of (i) WCC>30/µl or absence of CSF-restricted OCB and (ii) Q_Alb_>10×10^-3^ or absence of CSF-restricted OCB, both with simultaneous absence of positive MRZR, showed significant relevance (both with PLR=12.6, NLR=0.14) with the second lowest NLR overall. Thus, whereas positive MRZR can be used as a rule-out parameter for MOGAD, the absence of CSF-restricted oligoclonal bands, possibly combined with moderate-grade WCC elevation or moderate or severe dysfunction of the BSCFB can be used as a rule-in parameter for MOGAD.

A particularly interesting comparison in clinical practice is the one between MOGAD and MS patients without evidence of CSF-restricted OCB. As a complicating factor, positive MRZR is rare in OCB-negative patients and thus plays a significantly smaller role in the diagnosis of MS in these cases (45). While there is no statistically significant difference due to the small numbers of patients with OCB-negative MS in our cohort, we believe it is worthwhile to consider the direction in which the results point here: Pleocytosis, especially with intermediate cell count elevation, appears to be a typical finding in MOGAD patients, and cell counts above 15/µl did not occur within our MS patients without CSF-restricted OCB. In addition, BCSFB dysfunction was more than twice as frequent in MOGAD patients as in MS patients. No trends were apparent with respect to the humoral immune response. Of particular interest, positive MRZR was absent in both MOGAD and MS patients without CSF-restricted OCB. Unfortunately, data on cell differentiation were not available in this subcohort. In our opinion, a re-examination of these results in a larger cohort is necessary, but could prove very helpful.

Optic neuritis is a common symptom in both MOGAD and MS. While bilateral optic neuritis is an exception in MS (46), it can be a typical feature in MOGAD and occurs in up to 58% (47). In MOGAD, the optic nerve is typically affected in the proximal part and in a longitudinally extensive manner, and concomitant optic disc swelling, involvement of perineural tissue or moderate to severe edema may occur, whereas short-extent and peripheral involvement is particularly typical in MS (46, 48). While initial loss of visual acuity appears to be more severe in MOGAD, usually there is a good recovery in both diseases following corticosteroid treatment (46). Many papers have addressed the clinical and radiological differences of optic neuritis between the two disease entities (15, 48, 49), but systematic analysis of differences in CSF findings in these subcohorts is scarce. According to the current literature, optic neuritis in MOGAD presents with a normal WCC in up to two third of the cases, but can also show moderate pleocytosis and even a WCC>100/µl (16). In our cohort, MOGAD patients showed a normal WCC more frequently than MS patients, whereas in case of pleocytosis, a moderate WCC increase >30/μl was more frequent in MOGAD. Both results are thus compatible with previous work. The intrathecal synthesis of IgG, whether detected in the Reiber diagram or by detection of CSF-restricted OCB, as well as a positive MRZR, which occurred exclusively in MS patients, appear to be the best parameters to distinguish both diseases. Based on our results on the significant differences in cell differentiation between MOGAD and MS, the detection of neutrophils and plasma cells, respectively, could be helpful in clinical practice, but due to lack of data, we could not perform this analysis in the context of optic neuritis. The small number of patients limits the results of our analysis, and a larger systematic analysis is needed to confirm and further elaborate on these findings.

In summary, a number of useful pieces of information can be obtained from routine CSF clinical diagnostics to differentiate MOGAD from MS. Absence of CSF-restricted OCB and presence of moderate blood CSF barrier dysfunction stood out as the most relevant rule-in parameters for MOGAD in this context, while positive MRZR is confirmed as by far the best rule-out parameter for MOGAD. While circumstances such as relapse, remission, and clinical phenotype have a crucial impact on routine CSF parameters, a positive MRZR is now considered a robust marker to reliably distinguish MS from MOGAD regardless of the clinical context and time point. We consider it worthwhile to verify the results of this work in other and larger cohorts.

Our study is limited by its retrospective nature, the fact that it was conducted at a single center and the overall small number of MOGAD patients. MOG antibodies, which are detected in 3-5% of MOGAD patients only in CSF and not in the serum (50–52), have not been measured in CSF in our center, but could add new information to the cohort. Furthermore, our work is limited to routine clinical diagnostics that are ubiquitously available in laboratories and does not include emerging biomarkers such as neurofilament light-chain, glial fibrillary acidic protein, myelin basic protein, or even cytokine profiles from serum and CSF, which could help differentiate both disease entities at a higher level. On the plus side, we provide robust likelihood ratios for single and combined CSF parameters that are easy to use in clinical routine practice.

## Data availability statement

The original contributions presented in the study are included in the article/**Supplementary Material**. Further inquiries can be directed to the corresponding author.

## Ethics statement

The studies involving humans were approved by Informed consent was obtained from all patients or relatives. Since data of all patients were anonymized for this study, the local Cantonal Ethics Committee stated that the research project does not fall within the scope of the Human Reseach Act (HRA) and therefore, an authorization from the ethics committee is not required (BASEC Nr. Req-2022-01134). The studies were conducted in accordance with the local legislation and institutional requirements. Written informed consent for participation was not required from the participants or the participants’ legal guardians/next of kin in accordance with the national legislation and institutional requirements.

## Author contributions

BV had a major role in the acquisition of the data, analyzed and interpreted the data, and drafted the manuscript for intellectual content. IR, SN, MH, AL and AR acquired, analyzed and interpreted data, and revised the manuscript for intellectual content. DL, CH and AE interpreted data and revised the manuscript for intellectual content. MZ acquired and analyzed data and revised the manuscript for intellectual content. MR analyzed and interpreted data and revised the manuscript for intellectual content. IJ designed and conceptualized the study, acquired, analyzed and interpreted the data, and drafted the manuscript for intellectual content. All authors contributed to the article and approved the submitted version.
