# Supplementary material for: Basic CSF parameters and MRZ reaction help in differentiating MOG antibody-associated autoimmune disease versus multiple sclerosis
Source: Front Immunol. 2023 Sep 6;14:1237149. doi: 10.3389/fimmu.2023.1237149 (PMC10516557; doi:10.3389/fimmu.2023.1237149)

Supplementary Material

Basic CSF parameters and MRZ reaction help in differentiating MOG antibody-associated autoimmune disease versus multiple sclerosis

Benjamin Vlad, Ina Reichen, Stephan Neidhart, Marc Hilty, Dimitra Lekaditi, Christine Heuer, Amanda Eisele, Mario Ziegler, Markus Reindl, Andreas Lutterotti, Axel Regeniter, Ilijas Jelcic*

*** Correspondence:** Corresponding Author: ilijas.jelcic@uzh.ch

Supplementary Table 1. MOG antibody titers in MOGAD patients as measured using assay A and/or assay B. Assay A was a commercial kit using a fixed cell-based assay (Euroimmun, Kriens, Switzerland). Assay B was a live cell-based assay (Live CBA-IF, IgG(H+L) + Fc) quantified by immunofluorescence and end-point titration (Reindl et al., 2020) and performed at the Neurological Routine and Research Laboratory, Clinical Department of Neurology of the Medical University of Innsbruck (M. Reindl). Cut-off titer for MOG antibody positivity was ≥1:10 in assay A and ≥1:160 in assay B, respectively. Cut-off titer for high-titer MOG antibody levels (“clear positive”) was ≥1:320 in assay A and ≥1:640 in assay B, respectively.

MOGAD patient	Assay A	Assay B	MOGAD patient	Assay A	Assay B
MOGAD-01	1:320*	n.d.	MOGAD-16	1:32	1:640*
MOGAD-02	1:320*	1:5120*	MOGAD-17	n.d.	1:640*
MOGAD-03	1:32	1:1280*	MOGAD-18	1:100	1:640*
MOGAD-04	n.d.	1:5120*	MOGAD-19	1:32	1:160
MOGAD-05	n.d.	1:320	MOGAD-20	1:100	1:1280*
MOGAD-06	negative	1:640*	MOGAD-21	1:320*	1:640*
MOGAD-07	negative	1:160	MOGAD-22	negative	1:320
MOGAD-08	n.d.	1:640*	MOGAD-23	1:320*	n.d.
MOGAD-09	1:1000*	1:5120*	MOGAD-24	1:32	1:160
MOGAD-10	n.d.	1:10240*	MOGAD-25	n.d.	1:160
MOGAD-11	n.d.	1:160	MOGAD-26	n.d.	1:320
MOGAD-12	n.d.	1:1280*	MOGAD-27	n.d.	1:320
MOGAD-13	n.d.	1:640*	MOGAD-28	n.d.	1:320
MOGAD-14	n.d.	1:5120*	MOGAD-29	1:100	1:2560*
MOGAD-15	1:32	1:160	MOGAD-30	1:1000*	n.d.

n.d. – not determined
*high-titer MOG antibody levels as defined in assay A and/or assay B, respectively

Supplementary Table 2. Prevalences of positive MOG antibody resuults in MOGAD and MS patients using assay A and/or B. Assay A was a commercial kit using a fixed cell-based assay (Euroimmun, Kriens, Switzerland). Assay B was a live cell-based assay (Live CBA-IF, IgG(H+L) + Fc) quantified by immunofluorescence and end-point titration (Reindl et al., 2020) and performed at the Neurological Routine and Research Laboratory, Clinical Department of Neurology of the Medical University of Innsbruck (M. Reindl). Cut-off titer for MOG antibody positivity was ≥1:10 in assay A and ≥1:160 in assay B, respectively. Cut-off titer for high-titer MOG antibody levels ("clear positive") was ≥1:320 in assay A and ≥1:640 in assay B, respectively.

	MOGAD	MS
Assay A		
- Positive MOG antibody results in assay A (≥1:10), i.e. including high-titer and positive low-titer results	14/17 (82.4%)	1/89 (1.1%)*
- Clear positive (i.e. high-titer) MOG antibody results in assay A (≥1:320)	6/17 (35.3%)	0/89 (0.0%)
- Positive low-titer MOG antibody results in assay A (≥1:10, but <1:320)	8/17 (47.1%)	1/89 (1.1%)*
Assay B		
- Positive MOG antibody results in assay B (≥1:160), i.e. including high-titer and positive low-titer results	27/27 (100.0%)	0/23 (0.0%)
- Clear positive (i.e. high-titer) MOG antibody results in assay B (≥1:640)	16/27 (59.3%)	0/23 (0.0%)
- Positive low-titer MOG antibody results in assay B (≥1:160 but <1:640)	11/27 (40.7%)	0/23 (0.0%)
Assay A and B combined		
- Positive MOG antibody results in assay A (≥1:10) or in assay B (≥1:160), i.e. including high-titer and positive low-titer results	30/30 (100.0%)	1/97 (1.0%)*
- Clear positive (i.e. high-titer) MOG antibody results in assay A (≥1:320) or in assay B (≥1:640)	18/30 (60.0%)	0/97 (0.0%)
- Positive low-titer MOG antibody results in assay A (≥1:10, but <1:320) and/or assay B (≥1:160 but <1:640)	11/30 (36.7%)	1/97 (1.0%)*

* 1 MS patient with MOG antibody titer 1:32 in assay A, this patient tested negative in assay B.

Supplementary Table 3. Prevalence of diagnostic criteria fulfilled and typical MRI findings in MOGAD versus MS patients.

	MOGAD	MS
Diagnostic criteria for MOGAD fulfilled (Banwell et al., 2023)	30/30 (100.0%)	0/189 (0.0%)
Diagnostic criteria for MS fulfilled (Thompson et al., 2018)	0/30 (0.0%)	189/189 (100.0%)
Radiological findings typical of MOGAD (Banwell et al., 2023)	28/30* (93.3%)	0/189 (0.0%)
Radiological findings typical of MS (Filippi et al., 2019;Wattjes et al., 2021)	3/30** (10.0%)	189/189 (100.0%)

*2 MOGAD patients with ON did not fulfill radiological criteria for MOGAD in the first brain MRI, but they fulfilled diagnostic criteria for MOGAD and showed clear positive MOG antibody titers.

**3 MOGAD patients with longitudinally extensive transverse myelitis (LETM) fulfilled diagnostic criteria for MOGAD and showed MOGAD-typical radiological findings, but also fulfilled radiological, but not diagnostic, criteria for MS (inflammatory lesions with dissemination in time and space). 2 of those 3 patients showed no CSF-restricted OCB.

References:

Banwell, B., Bennett, J.L., Marignier, R., Kim, H.J., Brilot, F., Flanagan, E.P., Ramanathan, S., Waters, P., Tenembaum, S., Graves, J.S., Chitnis, T., Brandt, A.U., Hemingway, C., Neuteboom, R., Pandit, L., Reindl, M., Saiz, A., Sato, D.K., Rostasy, K., Paul, F., Pittock, S.J., Fujihara, K., and Palace, J. (2023). Diagnosis of myelin oligodendrocyte glycoprotein antibody-associated disease: International MOGAD Panel proposed criteria. *Lancet Neurol* 22**,** 268-282.

Filippi, M., Preziosa, P., Banwell, B.L., Barkhof, F., Ciccarelli, O., De Stefano, N., Geurts, J.J.G., Paul, F., Reich, D.S., Toosy, A.T., Traboulsee, A., Wattjes, M.P., Yousry, T.A., Gass, A., Lubetzki, C., Weinshenker, B.G., and Rocca, M.A. (2019). Assessment of lesions on magnetic resonance imaging in multiple sclerosis: practical guidelines. *Brain* 142**,** 1858-1875.

Thompson, A.J., Banwell, B.L., Barkhof, F., Carroll, W.M., Coetzee, T., Comi, G., Correale, J., Fazekas, F., Filippi, M., Freedman, M.S., Fujihara, K., Galetta, S.L., Hartung, H.P., Kappos, L., Lublin, F.D., Marrie, R.A., Miller, A.E., Miller, D.H., Montalban, X., Mowry, E.M., Sorensen, P.S., Tintore, M., Traboulsee, A.L., Trojano, M., Uitdehaag, B.M.J., Vukusic, S., Waubant, E., Weinshenker, B.G., Reingold, S.C., and Cohen, J.A. (2018). Diagnosis of multiple sclerosis: 2017 revisions of the McDonald criteria. *Lancet Neurol* 17**,** 162-173.

Wattjes, M.P., Ciccarelli, O., Reich, D.S., Banwell, B., de Stefano, N., Enzinger, C., Fazekas, F., Filippi, M., Frederiksen, J., Gasperini, C., Hacohen, Y., Kappos, L., Li, D.K.B., Mankad, K., Montalban, X., Newsome, S.D., Oh, J., Palace, J., Rocca, M.A., Sastre-Garriga, J., Tintoré,

M., Traboulsee, A., Vrenken, H., Yousry, T., Barkhof, F., Rovira, À; Magnetic Resonance Imaging in Multiple Sclerosis study group; Consortium of Multiple Sclerosis Centres; North American Imaging in Multiple Sclerosis Cooperative MRI guidelines working group (2021). 2021 MAGNIMS-CMSC-NAIMS consensus recommendations on the use of MRI in patients with multiple sclerosis. *Lancet Neurol* 20**,** 653-670.

Supplementary Table 4. Comparison of basic CSF parameters in MOGAD patients with monophasic and relapsing disease course.

	**Overall**	**Monophasic disease course**	**Relapsing disease course**	**p-value**
Pleocytosis, n/N (%)	12/30 (40.0%)	5/16 (31.3%)	7/14 (50.0%)	0.457
- WCC, mean (SD)	36.1 (95.2)	28.4 (53.0)	18.3 (25.4)	0.660
- WCC>15/µl, n (%)	10/30 (33.3%)	5/16 (31.3%)	5/14 (35.7%)	1.000
- WCC>30/µl, n (%)	6/30 (20.0%)	3/16 (18.8%)	3/14 (21.4%)	1.000
Elevated Q_{Alb}, n/N (%)	13/30 (43.3%)	8/16 (50.0%)	5/14 (35.7%)	0.484
IgG_{IF}>0%, n/N (%)	4/30 (13.3%)	2/16 (12.5%)	2/14 (14.3%)	1.000
IgA_{IF}>0%, n/N (%)	0/30 (0.0%)	0/16 (0.0%)	0/14 (0.0%)	-
IgM_{IF}>0%, n/N (%)	4/30 (13.3%)	2/16 (12.5%)	2/14 (14.3%)	1.000
CSF-restricted OCB, n/N (%)	5/30 (16.7%)	2/16 (12.5%)	3/14 (21.4%)	0.642
Positive MRZR[1], n/N (%)	0/30 (0.0%)	0/16 (0.0%)	0/14 (0.0%)	-
Elevated CSF lactate, n/N (%)	3/28 (10.7%)	1/16 (6.3%)	2/12 (16.7%)	0.560
Pathologic CSF/serum glucose ratio, n/N (%)	4/28 (14.3%)	3/16 (18.8%)	1/12 (8.3%)	0.613

[1]positive MRZ reaction (MRZR) was defined as intrathecal production of IgGs reactive against at least two of three antigens, measles (M), rubella (R) and varicella zoster (Z) virus antigens, i.e, M+R or M+Z or R+Z or M+R+Z.

Supplementary Table 5. Review of basic CSF parameters in MOGAD patients with optic neuritis (ON) and myelitis (MY) as first clinical presentation (A) in the current study, (B) in the study of Jarius et al., 2020b*, and (C) in both cohorts combined.

	**A) current study**				**B) Jarius et al., 2020b***				**C) both cohorts combined**			
	**Overall**	**ON**	**MY**	**p-value**	**Overall**	**ON**	**MY**	**p-value**	**Overall**	**ON**	**MY**	**p-value**
Pleocytosis, n/N (%)	12/30 (40.0%)	6/21 (28.6%)	6/9 (66.7%)	0.102	64/107 (59.8%)	18/53 (34.0%)	46/54 (85.2%)	< **0.001**		24/74 (32.4%)	52/63 (82.5%)	< **0.001**
WCC, mean (SD)	36.1 (95.2)	9.8 (20.0)	55.7 (60.3)	**0.007**	-	-	-	-	-	-	-	-
WCC>100/µl, n/N (%)	3/30 (10.0%)	0/21 (0.0%)	3/9 (33.3%)	< **0.001**	18/106 (17.0%)	1/52 (1.9%)	17/54 (31.5%)	< **0.001**	21/136 (15.4%)	1/73 (1.4%)	20/63 (31.7%)	< **0.001**
Elevated Q_{Alb}, n/N (%)	13/30 (43.3%)	8/21 (28.6%)	5/9 (55.6%)	0.443	44/96 (45.8%)	16/46 (34.8%)	28/50 (56.0%)	**0.043**	57/126 (45.2%)	24/67 (35.8%)	33/59 (55.9%)	**0.031**
IgG_{IF}>0% (=Q_{IgG}>Q_{lim}(IgG)), n/N (%)	4/30 (13.3)	3/21 (14.3%)	1/9 (11.1%)	1.000	8/91 (8.8%)	2/44 (4.5%)	6/47 (12.8%)	0.269	12/121 (9.9%)	5/65 (7.7%)	7/56 (12.5%)	0.543
IgA_{IF}>0% (=Q_{IgA}>Q_{lim}(IgA)), n/N (%)	0/30 (0.0%)	0/21 (0.0%)	0/9 (0.0%)	-	4/76 (5.3%)	2/38 (5.3%)	2/38 (5.3%)	1.000	4/106 (3.8%)	2/59 (3.4%)	2/47 (4.3%)	1.000
IgM_{IF}>0% (=Q_{IgM}>Q_{lim}(IgM)), n/N (%)	4/30 (13.3%)	1/21 (4.8%)	3/9 (33.3%)	0.069	12/77 (15.6%)	3/39 (8.0%)	9/38 (24.0%)	0.065	16/107 (15.0%)	4/60 (6.7%)	12/47 (25.5%)	**0.012**
CSF-restricted OCB, n/N (%)	5/30 (16.7%)	3/21 (14.3%)	2/9 (22.2%)	0.622	10/100 (10.0%)	3/50 (6.0%)	7/50 (14.0%)	0.317	15/130 (11.5%)	6/71 (8.5%)	9/59 (15.3%)	0.276
Positive MRZR, n/N (%)	0/30 (0.0%)	0/21 (0.0%)	0/9 (0.0%)	-	0/62 (0.0%)	-	-	-	0/92 (0.0%)	-	-	-
Elevated CSF lactate, n/N (%)	3/28 (10.7%)	1/19 (5.3%)	2/9 (22.2%)	0.234	25/76 (32.9%)	7/39 (17.9%)	18/37 (48.6%)	**0.007**	28/104 (26.9%)	8/58 (13.8%)	20/46 (43.5%)	**0.001**
Pathologic CSF/serum glucose ratio, n/N (%)	4/28 (14.3%)	2/19 (10.5%)	2/9 (22.2%)	0.574	-	-	-	-	-	-	-	-

* Jarius, S., Pellkofer, H., Siebert, N., Korporal-Kuhnke, M., Hummert, M.W., Ringelstein, M., Rommer, P.S., Ayzenberg, I., Ruprecht, K., Klotz, L., Asgari, N., Zrzavy, T., Hoftberger, R., Tobia, R., Buttmann, M., Fechner, K., Schanda, K., Weber, M., Asseyer, S., Haas, J., Lechner, C., Kleiter, I., Aktas, O., Trebst, C., Rostasy, K., Reindl, M., Kumpfel, T., Paul, F., Wildemann, B., and In Cooperation with the Neuromyelitis Optica Study, G. (2020b). Cerebrospinal fluid findings in patients with myelin oligodendrocyte glycoprotein (MOG) antibodies. Part 1: Results from 163 lumbar punctures in 100 adult patients. *J Neuroinflammation* 17**,** 261.

Supplementary Table 6. Mean values of parameters indicating intrathecal production of IgG, IgA or IgM according to Reiber's diagram.

Parameter	Overall	MOGAD	MS	p-value
**A) Mean CSF/serum quotient of IgG, IgA or IgM**				
mean Q_{IgG} (SD)	4.4 (2.8)	4.6 (4.4)	4.4 (2.5)	0.285
mean Q_{IgA} (SD)	1.8 (1.8)	2.5 (2.7)	1.7 (1.7)	0.024
mean Q_{IgM} (SD)	0.9 (1.6)	1.3 (2.5)	0.8 (1.4)	0.771
**B) Mean intrathecal fraction of IgG in patients with intrathecal production of IgG (IgG_{IF}>0%)**				
mean IgG_{IF} (SD), if IgG_{IF}>0%	38.5 (18.6)	31.1 (23.9)	38.8 (18.4)	0.617

Supplementary Table 7. Frequency of patients with intrathecal antiviral IgG production. Intrathecal production of IgG against a specific viral antigen is present, if CSF/serum antibody index (CAI) is ≥ 1.5. Frequencies are shown for the single virus-specific antibody reactivity species, measles (M), rubella (R) and varicella zoster (Z) virus antigens, and the combinations of these (M+R, R+Z, M+Z and M+R+Z).

**Parameter**	**Overall**	**MOGAD**	**MS**	**p-value**
0/3 antiviral CAI values>1.5, n/N (%)	82/219 (40.6%)	24/30 (80.0%)	65/189 (34.5%)	**<0.001**
1/3 antiviral CAI values>1.5 (M or R or Z), n/N (%)	63/219 (28.8%)	6/30 (20.0%)	57/189 (30.2%)	0.287
2/3 antiviral CAI values>1.5 (M+R or R+Z or M+Z), n/N (%)	37/219 (16.9%)	0/30 (0.0%)	37/189 (19.6%)	**0.003**
3/3 antiviral CAI values>1.5 (M+R+Z), n/N (%)	30/219 (13.7%)	0/30 (0.0%)	30/189 (15.9%)	**0.018**

Supplementary Table 8. Mean values of measles (M)-, rubella (R)- and zoster (Z)-specific CSF/serum antibody index (CAI) values. Intrathecal production of IgG against a specific viral antigen is present, if CSF/serum antibody index (CAI) is ≥ 1.5

**Parameter**	**Overall**	**MOGAD**	**MS**	**p-value**
Measles (M)-specific CAI, mean (SD)	2.1 (6.1)	1.2 (1.8)	2.2 (5.8)	0.066
Rubella (R)-specific CAI, mean (SD)	2.2 (3.4)	1.0 (0.5)	2.5 (3.6)	**< 0.001**
Zoster (Z)-specific CAI, mean (SD)	2.9 (5.4)	1.1 (0.5)	3.0 (5.1)	**< 0.001**

Supplementary Table 9. Review of frequencies of elevated basic CSF parameters in patients with MOGAD among studies reported in the current literature. The frequencies elevated CSF white cell count (WCC), elevated CSF protein or elevated Q_{Alb}, and CSF-specific oligoclonal bands (OCB) indicating intrathecal immunoglobulin synthesis are shown.

**Study (reference)**	**Cohort, pediatric and/or adult**	**CSF WCC elevated, n/N (%)**	**CSF protein or Q_{Alb} elevated, n/N (%)**	**CSF-specific OCB, n/N (%)**
Rostasy et al., 2012	pediatric	n.r.	n.r.	4/17 (23.5%)
Huppke et al., 2013	pediatric	6/7 (85.7%)	n.r.	0/7 (0.0%)
Hacohen et al., 2015	pediatric	8/19* (42.1%)	5/16 (31.3%)	1/16 (6.3%)
Jurynczyk et al., 2017	pediatric (n=22) and adult (n=53)	18/47 (38.3%)	23/50 (46.0%)	7/57 (12.3%)
Jarius et al., 2020a	pediatric	56/103 (54.4%)	36/79 (45.6%)	11/96 (11.5%)
Jarius et al., 2020b	adult	82/159 (51.6%)	67/139 (48.2%)	20/151 (13.2%)
Current work	adult	12/30 (40.0%)	13/30 (43.0%)	5/30 (16.7%)
Overall	pediatric and adult	182/365 (49.9%)	144/314 (45.9%)	48/374 (12.8%)

* WCC>10/µl

References:

Hacohen, Y., Absoud, M., Deiva, K., Hemingway, C., Nytrova, P., Woodhall, M., Palace, J., Wassmer, E., Tardieu, M., Vincent, A., Lim, M., and Waters, P. (2015). Myelin oligodendrocyte glycoprotein antibodies are associated with a non-MS course in children. *Neurol Neuroimmunol Neuroinflamm* 2**,** e81.

Huppke, P., Rostasy, K., Karenfort, M., Huppke, B., Seidl, R., Leiz, S., Reindl, M., and Gartner, J. (2013). Acute disseminated encephalomyelitis followed by recurrent or monophasic optic neuritis in pediatric patients. *Mult Scler* 19**,** 941-946.

Jarius, S., Lechner, C., Wendel, E.M., Baumann, M., Breu, M., Schimmel, M., Karenfort, M., Marina, A.D., Merkenschlager, A., Thiels, C., Blaschek, A., Salandin, M., Leiz, S., Leypoldt,

F., Pschibul, A., Hackenberg, A., Hahn, A., Syrbe, S., Strautmanis, J., Hausler, M., Krieg, P., Eisenkolbl, A., Stoffels, J., Eckenweiler, M., Ayzenberg, I., Haas, J., Hoftberger, R., Kleiter, I., Korporal-Kuhnke, M., Ringelstein, M., Ruprecht, K., Siebert, N., Schanda, K., Aktas, O., Paul, F., Reindl, M., Wildemann, B., Rostasy, K., In Cooperation with The, B.S.G., and The Neuromyelitis Optica Study, G. (2020a). Cerebrospinal fluid findings in patients with myelin oligodendrocyte glycoprotein (MOG) antibodies. Part 2: Results from 108 lumbar punctures in 80 pediatric patients. *J Neuroinflammation* 17**,** 262.

Jarius, S., Pellkofer, H., Siebert, N., Korporal-Kuhnke, M., Hummert, M.W., Ringelstein, M., Rommer, P.S., Ayzenberg, I., Ruprecht, K., Klotz, L., Asgari, N., Zrzavy, T., Hoftberger, R., Tobia, R., Buttmann, M., Fechner, K., Schanda, K., Weber, M., Asseyer, S., Haas, J., Lechner, C., Kleiter, I., Aktas, O., Trebst, C., Rostasy, K., Reindl, M., Kumpfel, T., Paul, F., Wildemann, B., and In Cooperation with the Neuromyelitis Optica Study, G. (2020b). Cerebrospinal fluid findings in patients with myelin oligodendrocyte glycoprotein (MOG) antibodies. Part 1: Results from 163 lumbar punctures in 100 adult patients. *J Neuroinflammation* 17**,** 261.

Jurynczyk, M., Messina, S., Woodhall, M.R., Raza, N., Everett, R., Roca-Fernandez, A., Tackley, G., Hamid, S., Sheard, A., Reynolds, G., Chandratre, S., Hemingway, C., Jacob, A., Vincent, A., Leite, M.I., Waters, P., and Palace, J. (2017). Clinical presentation and prognosis in MOG-antibody disease: a UK study. *Brain* 140**,** 3128-3138.

Rostasy, K., Mader, S., Schanda, K., Huppke, P., Gartner, J., Kraus, V., Karenfort, M., Tibussek, D., Blaschek, A., Bajer-Kornek, B., Leitz, S., Schimmel, M., Di Pauli, F., Berger, T., and Reindl, M. (2012). Anti-myelin oligodendrocyte glycoprotein antibodies in pediatric patients with optic neuritis. *Arch Neurol* 69**,** 752-756.

Supplementary Table 10. Review of frequencies of positive single measles (M)-, rubella (R)- and varicella zoster virus (Z)-specific CSF/serum antibody index (CAI) and of positive MRZ reaction (MRZR)[1] among samples from patients with MOGAD reported in the current literature.

**Study (reference)**	**Cohort, pediatric and/or adult**	**M-AI positive, n/N (%)**	**R-AI positive, n/N (%)**	**Z-AI positive, n/N (%)**	**MRZR positive[1] n/N (%)**
Jarius et al., 2020a	pediatric	0/25 (0.0%)	0/19 (0.0%)	0/28 (0.0%)	0/28 (0.0%)
Jarius et al., 2020b	adult	2/61 (3.3%)	1/52 (1.9%)	3/76 (3.9%)	0/62 (0.0%)
Current work	adult	2/30 (6.7%)	1/30 (0.0%)	0/30 (0.0%)	0/30 (0.0%)
Overall	adult and pediatric	4/116 (3.4%)	2/101 (2.0%)	3/134 (2.2%)	0/120 (0.0%)

[1]positive MRZ reaction (MRZR) was defined as intrathecal production of IgGs reactive against at least two of three antigens, measles (M), rubella (R) and varicella zoster (Z) virus antigens, i.e, M+R or M+Z or R+Z or M+R+Z.

References:

Jarius, S., Lechner, C., Wendel, E.M., Baumann, M., Breu, M., Schimmel, M., Karenfort, M., Marina, A.D., Merkenschlager, A., Thiels, C., Blaschek, A., Salandin, M., Leiz, S., Leypoldt, F., Pschibul, A., Hackenberg, A., Hahn, A., Syrbe, S., Strautmanis, J., Hausler, M., Krieg, P., Eisenkolbl, A., Stoffels, J., Eckenweiler, M., Ayzenberg, I., Haas, J., Hoftberger, R., Kleiter, I., Korporal-Kuhnke, M., Ringelstein, M., Ruprecht, K., Siebert, N., Schanda, K., Aktas, O., Paul, F., Reindl, M., Wildemann, B., Rostasy, K., In Cooperation with The, B.S.G., and The Neuromyelitis Optica Study, G. (2020a). Cerebrospinal fluid findings in patients with myelin oligodendrocyte glycoprotein (MOG) antibodies. Part 2: Results from 108 lumbar punctures in 80 pediatric patients. *J Neuroinflammation* 17**,** 262.

Jarius, S., Pellkofer, H., Siebert, N., Korporal-Kuhnke, M., Hummert, M.W., Ringelstein, M., Rommer, P.S., Ayzenberg, I., Ruprecht, K., Klotz, L., Asgari, N., Zrzavy, T., Hoftberger, R., Tobia, R., Buttmann, M., Fechner, K., Schanda, K., Weber, M., Asseyer, S., Haas, J., Lechner, C., Kleiter, I., Aktas, O., Trebst, C., Rostasy, K., Reindl, M., Kumpfel, T., Paul, F., Wildemann, B., and In Cooperation with the Neuromyelitis Optica Study, G. (2020b). Cerebrospinal fluid findings in patients with myelin oligodendrocyte glycoprotein (MOG) antibodies. Part 1: Results from 163 lumbar punctures in 100 adult patients. *J Neuroinflammation* 17**,** 261.

Supplementary Table 11. Review of (A) probable prevalence of MS and MOGAD, respectively, in Switzerland, (B) disease-specific sensitivity of a positive MOG antibody in MOGAD or positive MRZ reaction in MS, and (C) estimated population-based prevalence of the respective positive diagnostic test depending on probable prevalence of MS and MOGAD, respectively, in Switzerland, i.e. calculated by using respective parameters from (A) and (B).

**A)**			
**Parameter**	**MOGAD in general population**	**MS in general population**	**p-value**
Probable population-based prevalence of disease	2 / 100.000<br>(O'connell et al., 2017; Orlandi et al., 2022)	190 / 100.000<br>(Blozik et al., 2017)	< **0.00001**
**B)**			
**Parameter**	**MOG antibody in MOGAD**	**positive MRZ reaction in MS**	**p-value**
Disease-specific sensitivity of respective positive diagnostic test	100%<br>(Banwell et al., 2023; Reindl et al., 2020)	35.4%<br>(current work)	< **0.00001**
**C)**			
**Parameter**	**MOG antibody in general population**	**Positive MRZ reaction in general population**	**p-value**
Estimated population-based prevalence of the respective positive diagnostic test, calculated by using respective parameters in (A) and (B)	2 / 100.000	67.3 / 100.000	< **0.00001**

References:

Banwell, B., Bennett, J.L., Marignier, R., Kim, H.J., Brilot, F., Flanagan, E.P., Ramanathan, S., Waters, P., Tenembaum, S., Graves, J.S., Chitnis, T., Brandt, A.U., Hemingway, C., Neuteboom, R., Pandit, L., Reindl, M., Saiz, A., Sato, D.K., Rostasy, K., Paul, F., Pittock, S.J., Fujihara, K., and Palace, J. (2023). Diagnosis of myelin oligodendrocyte glycoprotein antibody-associated disease: International MOGAD Panel proposed criteria. *Lancet Neurol* 22**,** 268-282.

Blozik, E., Rapold, R., Eichler, K., and Reich, O. (2017). Epidemiology and costs of multiple sclerosis in Switzerland: an analysis of health-care claims data, 2011-2015. *Neuropsychiatr Dis Treat* 13**,** 2737-2745.

O'connell, K., Hamilton-Shield, A., Woodhall, M., Messina, S., Mariano, R., Waters, P., Ramdas, S., Leite, M.I., and Palace, J. (2020). Prevalence and incidence of neuromyelitis optica spectrum disorder, aquaporin-4 antibody-positive NMOSD and MOG antibody-positive disease in Oxfordshire, UK. *J Neurol Neurosurg Psychiatry* 91**,** 1126-1128.

Orlandi, R., Mariotto, S., and Gajofatto, A. (2022). Prevalence, incidence, and season distribution of MOG antibody-associated disease in the province of Verona, Italy. *Mult Scler Relat Disord* 63**,** 103884.

Reindl, M., Schanda, K., Woodhall, M., Tea, F., Ramanathan, S., Sagen, J., Fryer, J.P., Mills, J., Teegen, B., Mindorf, S., Ritter, N., Krummrei, U., Stocker, W., Eggert, J., Flanagan, E.P., Ramberger, M., Hegen, H., Rostasy, K., Berger, T., Leite, M.I., Palace, J., Irani, S.R., Dale, R.C., Probst, C., Probst, M., Brilot, F., Pittock, S.J., and Waters, P. (2020). International multicenter examination of MOG antibody assays. *Neurol Neuroimmunol Neuroinflamm* 7.

Supplementary Table 12. Impact of pretest probability on usefulness of MOG antibody test, given that population-based prevalence of MOGAD is 2 / 100.000 and of MS is 190 / 100.000 in Switzerland (see supplemental Table 8), and that positive MRZ reaction is found in 35% of MS patients and in 0% of MOGAD patients.

**A)** When used in 100 patients with a 50% likelihood of being positive (e.g., longitudinally extensive transverse myelitis), and when (**A1**) all patients with at least “low positive” (i.e. low-titer) MOG-specific antibody results in a cell-based assay are included as MOG-seropositive cases resulting in a sensitivity of 100% and a specificity of 95% (Banwell et al., 2023), or (**A2**) all patients with at least “clear positive” (i.e. high-titer) MOG-specific antibody results in a cell-based assay are included as MOG-seropositive cases resulting in a sensitivity of 60% and a specificity of 100% (Banwell et al., 2023).						
	**A1)** MOG-specific antibody at least “low positive”			**A2)** MOG-specific antibody at least “clear positive”		
	MOGAD	not MOGAD	Predictive values	MOGAD	not MOGAD	Predictive values
MOG-sero-positive	a) 50	b) 2.5	Positive predictive value [a/(a+b)]: 95%	a) 30	b) 0	Positive predictive value [a/(a+b)]: 100%
MOG-sero-negative	c) 0	d) 47.5	Negative predictive value [d/(c+d)]: 100%	c) 20	d) 50	Negative predictive value [d/(c+d)]: 71.5%
Sensitivity or specificity	Sensitivity [a/(a+c)]: 100.0%	Specificity [d/(b+d)]: 95.0%	**➔ 5% false positive tests**	Sensitivity [a/(a+c)]: 60.0%	Specificity [d/(b+d)]: 100.0%	**➔ 0% false positive tests**

**B)** When used in 100 patients with a 1% likelihood of being positive (i.e., all patients without longitudinally extensive transverse myelitis or multiple sclerosis), and when (**B1**) all patients with at least "low positive" (i.e. low-titer) MOG-specific antibody results in a cell-based assay are included as MOG-seropositive cases resulting in a sensitivity of 100% and a specificity of 95% (Banwell et al., 2023), or (**B2**) all patients with at least "clear positive" (i.e. high-titer) MOG-specific antibody results in a cell-based assay are included as MOG-seropositive cases resulting in a sensitivity of 60% and a specificity of 100% (Banwell et al., 2023).						
	**B1)** MOG-specific antibody at least "low positive"			**B2)** MOG-specific antibody at least "clear positive"		
	MOGAD	not MOGAD	Predictive values	MOGAD	not MOGAD	Predictive values
MOG-sero-positive	a) 1	b) 5	Positive predictive value [a/(a+b)]: 16.7%	a) 0.6	b) 0	Positive predictive value [a/(a+b)]: 100%
MOG-sero-negative	c) 0	d) 94	Negative predictive value [d/(c+d)]: 94%	c) 0.4	d) 99	Negative predictive value [d/(c+d)]: 99.6%
Sensitivity or specificity	Sensitivity [a/(a+c)]: 100.0%	Specificity [d/(b+d)]: 95.0%	**➔ 83.3% false positive tests**	Sensitivity [a/(a+c)]: 60.0%	Specificity [d/(b+d)]: 100.0%	**➔ 0% false positive tests**

**C)** When used in 100 patients with a 1.6% likelihood of being positive (i.e., all patients with negative MRZ reaction including multiple sclerosis), and when (**C1**) all patients with at least "low positive" (i.e. low-titer) MOG-specific antibody results in a cell-based assay are included as MOG-seropositive cases resulting in a sensitivity of 100% and a specificity of 95% (Banwell et al., 2023), or (**C2**) all patients with at least "clear positive" (i.e. high-titer) MOG-specific antibody results in a cell-based assay are included as MOG-seropositive cases resulting in a sensitivity of 60% and a specificity of 100% (Banwell et al., 2023).						
	**C1)** MOG antibody assay cut-off value: 1:160			**C2)** MOG antibody assay cut-off value: 1:640		
	MOGAD	not MOGAD	Predictive values	MOGAD	not MOGAD	Predictive values
MOG-sero-positive	a) 1.6	b) 4.9	Positive predictive value [a/(a+b)]: 24.6%	a) 1	b) 0	Positive predictive value [a/(a+b)]: 100%
MOG-sero-negative	c) 0	d) 93.5	Negative predictive value [d/(c+d)]: 100%	c) 0.64	d) 98.36	Negative predictive value [d/(c+d)]: 99.4%
Sensitivity or specificity	Sensitivity [a/(a+c)]: 100.0%	Specificity [d/(b+d)]: 95.0%	**➔ 75.4% false positive tests**	Sensitivity [a/(a+c)]: 60.0%	Specificity [d/(b+d)]: 100.0%	**➔ 0% false positive tests**

References:

Banwell, B., Bennett, J.L., Marignier, R., Kim, H.J., Brilot, F., Flanagan, E.P., Ramanathan, S., Waters, P., Tenembaum, S., Graves, J.S., Chitnis, T., Brandt, A.U., Hemingway, C., Neuteboom, R., Pandit, L., Reindl, M., Saiz, A., Sato, D.K., Rostasy, K., Paul, F., Pittock, S.J., Fujihara, K., and Palace, J. (2023). Diagnosis of myelin oligodendrocyte glycoprotein antibody-associated disease: International MOGAD Panel proposed criteria. *Lancet Neurol* 22**,** 268-282.

Supplementary Table 13. Comparison of basic CSF parameters in MOGAD and MS in patients without CSF-restricted OCB.

	**Overall**	**MOGAD**	**MS**	**p-value**
Pleocytosis, n (%)	11/36 (30.6%)	9/25 (36.0%)	2/11 (18.2%)	0.439
- WCC, mean (SD)	18.0 (40.7)	24.7 (45.7)	3.1 (2.7)	0.564
- WCC>15/µl, n/N (%)	7/36 (19.4%)	7/25 (28.0%)	0/11 (0.0%)	0.076
- WCC>30/µl, n/N (%)	5/36 (13.9%)	5/25 (20.0%)	0/11 (0.0%)	0.295
Elevated Q_{Alb}, n/N (%)	13/36 (36.1%)	11/25 (44.0%)	2/11 (18.2%)	0.259
IgG_{IF}>0%, n/N (%)	0/36 (0.0%)	0/25 (0.0%)	0/11 (0.0%)	-
IgA_{IF}>0%, n/N (%)	0/36 (0.0%)	0/25 (0.0%)	0/11 (0.0%)	-
IgM_{IF}>0%, n/N (%)	3/36 (8.3%)	2/25 (8.0%)	1/11 (9.1%)	1.000
Positve MRZR, n/N (%)	0/36 (0.0%)	0/25 (0.0%)	0/11 (0.0%)	-
Elevated CSF lactate, n/N (%)	5/34 (14.7%)	4/23 (17.4%)	1/11 (9.1%)	1.000
Pathologic CSF/serum glucose ratio	3/33 (9.1%)	3/23 (13.0%)	0/10 (0.0%)	0.536

Supplementary Figure 1. Comparison of MOG antibody titers between MOGAD and MS patients as measured using assay A and/or assay B. Assay A was a commercial kit using a fixed cell-based assay (Euroimmun, Kriens, Switzerland). Assay B was a live cell-based assay (Live CBA-IF, IgG(H+L) + Fc) quantified by immunofluorescence and end-point titration (Reindl et al., 2020) and performed at the Neurological Routine and Research Laboratory, Clinical Department of Neurology of the Medical University of Innsbruck (M. Reindl). Cut-off titer for MOG antibody positivity was ≥1:10 in assay A and ≥1:160 in assay B, respectively. Cut-off titer for high-titer MOG antibody levels ("clear positive") was ≥1:320 in assay A and ≥1:640 in assay B, respectively.

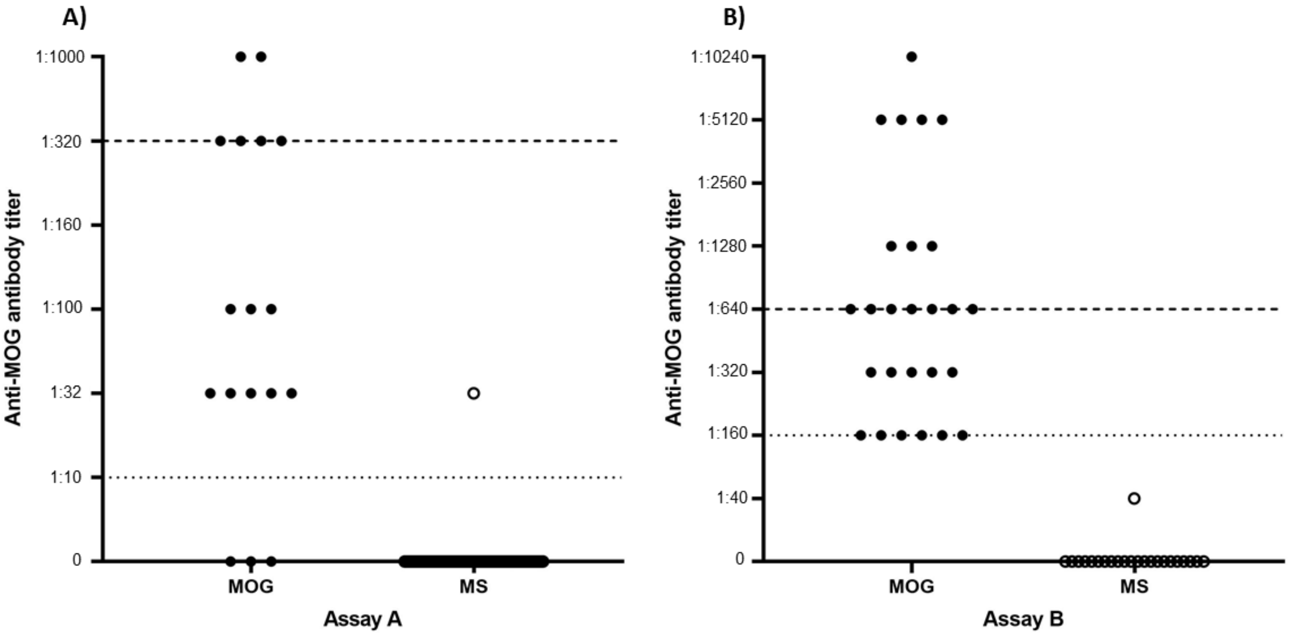

Supplementary Figure 2. A) Single dot distribution of WCC per µl in patients with MOGAD vs. MS. **B)** Single dot distribution of Q_{Alb} x 10^{-3} in patients with MOGAD vs. MS.

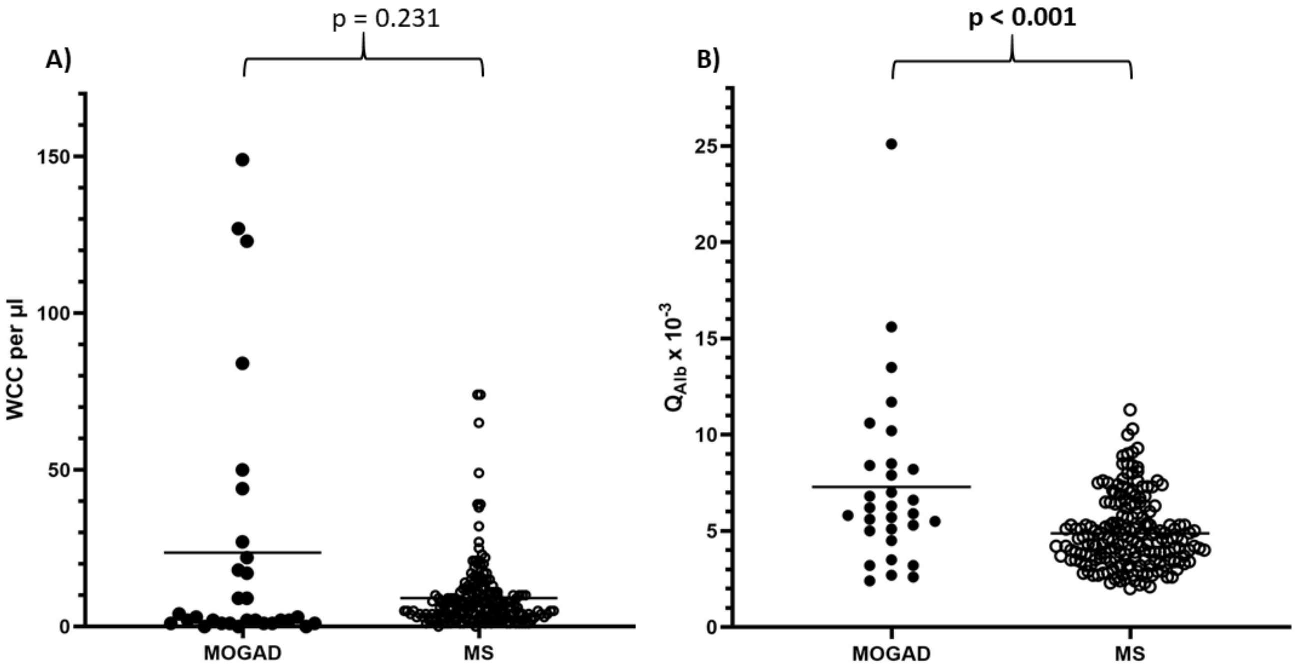

Supplement: Supplementary file 1 [file DataSheet_1.pdf]
